# New insights into early medieval Islamic cuisine: Organic residue analysis of pottery from rural and urban Sicily

**DOI:** 10.1371/journal.pone.0252225

**Published:** 2021-06-09

**Authors:** Jasmine Lundy, Lea Drieu, Antonino Meo, Viva Sacco, Lucia Arcifa, Elena Pezzini, Veronica Aniceti, Girolamo Fiorentino, Michelle Alexander, Paola Orecchioni, Alessandra Mollinari, Martin O. H. Carver, Oliver E. Craig

**Affiliations:** 1 Department of Archaeology, BioArCh, University of York, York, United Kingdom; 2 Dipartimento di Storia, Patrimonio Culturale, Formazione e Società, Università degli Studi di Roma Tor Vergata, Rome, Italy; 3 École française de Rome, Rome, Italy; 4 Facoltà di Scienze della Formazione, Università di Catania, Catania, Italy; 5 “Antonino Sallinas”, Regional Archaeological Museum of Palermo, Palermo, Italy; 6 Department of Natural History, University Museum of Bergen, Bergen, Norway; 7 Laboratory of Archaeobotany and Palaeoecology, Università del Salento, Lecce, Italy; Institucio Catalana de Recerca i Estudis Avancats, SPAIN

## Abstract

Sicily, during the 9^th^-12^th^ century AD, thrived politically, economically, and culturally under Islamic political rule and the capital of Palermo stood as a cultural and political centre in the Mediterranean Islamic world. However, to what extent the lifeways of the people that experienced these regimes were impacted during this time is not well understood, particularly those from lesser studied rural contexts. This paper presents the first organic residue analysis of 134 cooking pots and other domestic containers dating to the 9^th^ -12^th^ century in order to gain new insights into the culinary practices during this significant period. Ceramics from three sites in the urban capital of Palermo and from the rural town of Casale San Pietro were analysed and compared. The multi-faceted organic residue analysis identified a range of commodities including animal products, vegetables, beeswax, pine and fruit products in the ceramics, with a complex mixing of resources observed in many cases, across all four sites and ceramic forms. Alongside the identification of commodities and how they were combined, new light has been shed on the patterning of resource use between these sites. The identification of dairy products in calcite wares from the rural site of Casale San Pietro and the absence of dairy in ceramics from the urban centre of Palermo presents interesting questions regarding the role of rural sites in food consumption and production in Islamic Sicily. This is the first time organic residue analysis of ceramics has been used to explore foodways in a medieval multi-faith society and offers new pathways to the understanding of pottery use and resources that were prepared, consumed and combined, reflecting cuisine in different socio-economic environments within the pluralistic population of medieval Sicily.

## Introduction

Sicily is the largest Island in the Mediterranean Sea and is centrally located, between mainland Italy, North Africa and the Eastern Mediterranean. For several reasons including political connections, environmental and arable conditions, and not least its geographic location, the island of Sicily has been at the epicentre of political interest and subsequent conquests throughout ancient and medieval history. Of profound impact to the island, was the transition from a Byzantine political control (6^th^– 9^th^ century AD in western Sicily and 6^th^– 10^th^ in the eastern part of the Island) to Islamic rule (9^th^ to 11^th^ century AD and 10^th^ to 11^th^, in the west and east respectively) when Sicily became part of the dār al-Islām. In 827 AD the Aghlabid army landed in Mazara del Vallo, Trapani, located on the south-western coast of the island, following almost a century and a half of political unrest and regular raids from North Africa. In 831 AD Palermo was captured and since became the political and economic capital of Sicily. In the next two centuries, under the control of first the Aghlabid emirate (827–909 AD), then the Fatimid caliphate, ruled from Ifrīqiya, and then from Egypt, and finally the Kalbid emirate, who ruled the Island on behalf of the Fatimids from 948 AD, Palermo thrived politically, economically and culturally. Despite this, exactly how these Islamic political regimes impacted the lifeways of people in Palermo and more broadly elsewhere in Sicily, particularly in lesser studied rural areas, at this time is not fully understood.

Agriculture was undoubtedly at the heart of the Islamic economy but the degree to which Sicily was transformed by an Islamic “Green Revolution” requires careful evaluation. This term, originally coined by Watson [1, 2], refers to the association between the movement of Islamic communities and the introduction of new resources and agricultural innovations. Watson documents 17 plants that arrived in the Mediterranean from the Middle East with the Arabs, including spinach, cotton, durum wheat, sugar, citrus fruits and possibly broomcorn millet [1, 2]. Some aspects of Watson’s thesis have recently been revived, although not uncritically, both from historical research [3, 4] and from the development of archaeobotanical research [5–7]. Excavations at Mazara del Vallo (Trapani) have provided direct evidence of Watson’s ‘revolution plants’, including citrus fruits in 10th-11th century contexts and watermelon, aubergine, cotton, durum wheat and spinach during the 11th-13th centuries [8, 9]. There is no doubt that an advanced knowledge of agricultural and irrigation systems was introduced into Sicily during this time. However, in the absence of data from preceding contexts, the extent and impact of these innovations remain unclear. While the introduction of new plants is not contested, it has since been suggested that some resources were already present in the region, but gained new importance at the arrival of the Arabs [3]. Furthermore, evidence from written sources and ceramic studies suggest a more complex dynamic with regards to the production of agricultural commodities in Palermo. It has been suggested that an evolution in farming methods and an integration of Sicily into an Islamic botanic-food horizon is more related to the change of Palermo’s role and to the investments of the new elites [10, 11].

Alongside the agricultural economy, religion played an important and complex role in shaping the economy and consumption practices of Sicily during the early medieval period. Although under the Islamic political regime, Christian and Jewish communities remained in Sicily during the 9th-12th centuries, living alongside Muslims [12] (p.282). This complexity is often reduced to discussions of food taboos imposed by the Islamic religion. The consumption of pork and alcohol are prohibited in the hadiths but, to what extent these restrictions were observed in early medieval daily life is questionable, particularly in a pluralistic society as was Sicily at this time. For example, although generally found in low proportions, the discovery of pig remains found in Islamic contexts in Spain and Portugal, have been attributed to the presence of Christians or hunted wild boar [13, 14]. In terms of alcohol production and consumption, although prohibited by the hadith, wine may have been consumed by Christian and Jewish communities, or as Islamic medieval poems depict, by some members of the Muslim community also [15, 16]. The trade of wine to and from Islamic Sicily has also recently been observed by identifying organic biomarkers associated with grape products in amphorae [17].

Cuisine is a cultural phenomenon manifested in the way in which specific food resources are produced, procured, prepared, combined and consumed. By studying culinary practices in the archaeological record, it is possible to gain insight into the way that people both thought about and valued different foodstuffs and how food traditions and rituals were influenced by broader changes, such as in politics, economy, agricultural systems and religion [18–20]. Recent archaeobotanical [8, 9, 21] and faunal analysis [22, 23] from Islamic contexts in Sicily, have begun to address important questions regarding the resources that were available in Sicily under Islamic control. Written sources about Islamic cuisine have given insight into culinary practices across the Arabic-Islamic world [24–26]. However, distributed across a broad chronological period and geographic area, these writings mostly describe practices of élite consumption. In contrast, here we present the first large scale study of culinary practices in the early medieval Islamic world focusing on the chemical analysis of domestic cooking wares from rural and urban settlements. Unlike written sources, such evidence is more likely to capture everyday utilitarian practice and allow a more nuanced understanding of how foodstuffs were utilized in Islamic cuisine. By understanding pottery use we can gain a unique perspective of how resources were prepared, consumed and combined, in turn helping to answer important questions regarding daily life under Islamic political control in Sicily.

We applied well-established methods in organic residue analysis (ORA) [27–31] (S1 Text) to identify organic residues from 134 cooking pots and other domestic containers from three sites in Palermo and from the rural town of Casale San Pietro dating to the 9th-12th centuries AD (S1 Data). The research benefits from recent analysis of ceramics from this period that has succeeded in refining the chrono-typologies and identifying production centres [21, 32–36]. Only a small number of ceramics from medieval Sicily have previously been analysed using ORA [37]. Until now, investigations have been mostly limited to typological and petrographic studies, and virtually nothing is known about the use of such pottery in Sicily nor more broadly in early medieval Islamic society. To aid this analysis, and to further understand the contents of these ceramics, ORA was performed on a selection of modern vegetables thought to be present in Sicily during the Islamic regime (S3 Text). This study draws on evidence provided through archaeobotanical and faunal analysis, historical and contemporary accounts of Islamic-Arabic cuisine, and the identification of cooking wares.

With consideration of current ideas and evidence surrounding cuisine in early medieval Islamic societies, the focus of this research is two-fold: first we ask, how does the contents of these domestic containers contribute to our knowledge of cuisine in early medieval Islamic societies? Secondly, do the contents of these ceramics differ in urban and rural socio-economic settings?

## Sites and ceramics

### Palermo (Urban)

Pottery sherds (n = 83) were obtained from three sites in Palermo dating to the Islamic period; 24 from Castello San Pietro (CSP) [33], 26 from Gancia church (GA) [35] and 33 from Palazzo Bonagia (PB) [34] (S1 Data). Recent surveys at all three sites have enabled the identification of some of the earliest Islamic style pottery in Sicily. Strong typological links were made between tableware in the assemblage and those found in Aghlabid Ifrīqiya (located in Tunisia and part of Libya and Algeria), perhaps evidence of direct culinary links between Palermo and North Africa at this time [36]. In contrast, the vast majority of domestic cooking wares were produced in Palermo and appear typologically dissimilar to those found in Ifrīqiya [38]. As a major production site, Palermitan ceramics were distributed throughout the Island [32].

Castello San Pietro (CSP) is a site located adjacent to the harbour in Palermo. The earliest context of this excavation revealed an Islamic cemetery, which was succeeded by a cluster of houses constructed with little hiatus after the cemetery’s disuse [33]. One of the earliest closed contexts is US865, a well reused as a rubbish pit. The pottery from this context has been dated between the 9^th^ and the beginning of the 10^th^ century [33] (S1 Data). The sequence seen at CSP is repeated at the site of the Gancia church (GA), where burials in the Islamic rite are superseded by settlement (‘urbanisation’) with walls and midden heaps associated with pottery assigned to the end of the 9^th^–beginning of the 10^th^ century before the construction of the Fatimid citadel documented in 937 AD [35] (pp.197-200) [39] (p.339). The same horizon (late 9^th^/early 11^th^ century) has been noted at the excavations at Palazzo Bonagia (PB) [34] (p.226). The pottery at these sites, therefore, belongs to the early phase of Islamic Palermo (late 9^th^-early 10^th^ at CSP, GA and PB), and the first phase of Islamic urbanisation (10^th^- 11^th^ at GA and PB).

### Casale San Pietro (CLESP; rural)

**Fig 1:**
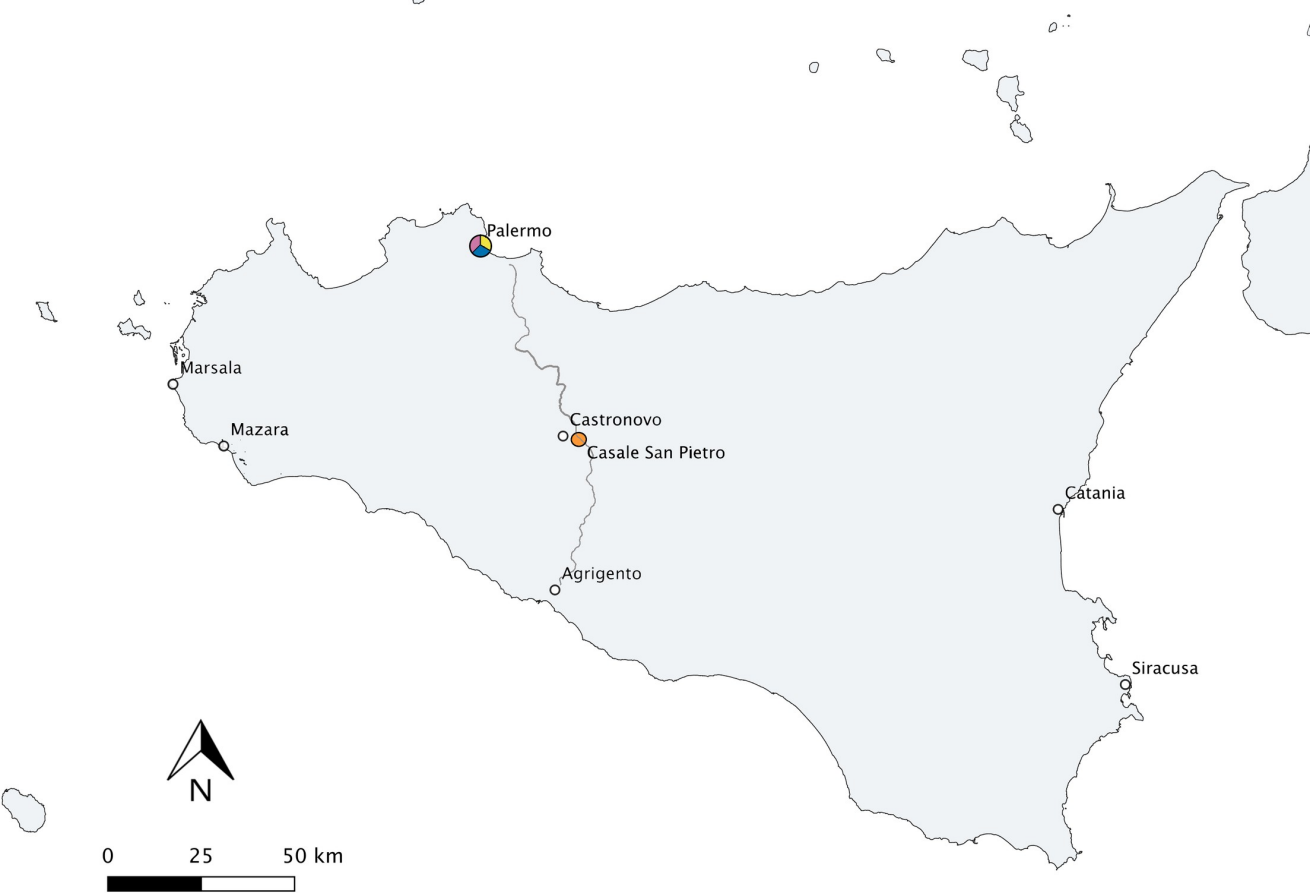
Map of Sicily showing the location of Palermo and Casale San Pietro. Colours represent the sites with ceramics used in this study where pink = CSP, yellow = GA, blue = PB and orange = CLESP. The main road between Palermo and Agrigento from North to South is marked on the map.

Pottery sherds (n = 51) were obtained from the site of Casale San Pietro (CLESP), located on the plain outside of the town of Castronovo di Sicilia in the centre of Sicily within the province of Palermo (Fig 1) (S1 Data). Excavations have revealed that the site was extensive and dedicated to agricultural production in 3-5^th^ century (a large *vicus* and a *statio*), continuing in a reduced form during the Byzantine period (6-9^th^ century), flourishing again during the Islamic period (especially in the 10-11^th^ century), and continuing but with a reduced economic profile in the 12/13^th^ century [20, 38, 39]. An impressive assemblage of ceramics has been identified from the 10^th^-12^th^ centuries. Whereas, from the 9^th^ century only a small number of cooking pots with unknown provenance have so far been identified. Analysis of the ceramic pastes of these ceramics has shown that the settlement relied heavily on pottery from Palermitan production at this time, depicting a strong link between this rural settlement and the capital of Palermo; likely one of economic reliance [21]. This raises interesting questions regarding the use of these imported ceramics vessels for local culinary uses in the rural settlement. A collection of handmade/slow thrown cooking pots and wheel thrown calcite ware, of unknown provenance but likely imported from elsewhere, were also identified.

### Ceramic corpus

Two main shapes of vessels suitable for use on fire have been distinguished, which correspond to at least two different technologies. Here we refer to ‘olla’ as vessel shapes that are slightly closed and deeper than wide in capacity and often have handles and usually a curved base. We refer to ‘cooking pots’ here as vessels that are more wide than deep and generally have a flat base and straighter sides. In addition, some additional forms were analysed: pans, stone plates, so called ‘braziers’ and lids (S1 Data). Ceramics from CSP and PB were obtained from the Soprintendenza di Palermo Storage Rooms at Via Magione, 44, 90133 Palermo and ceramics from GA were obtained from Soprintendenza di Palermo Storage Rooms located in the church of Santa Maria della Gancia, Via Alloro, 13, 90133 Palermo. Inventory numbers and sample numbers of the original studies of the ceramics from Palermo are referenced in (S1 Data). Samples from CLESP were obtained from the Comune di Castronovo storage rooms, located in Palazzo Giandalia, Via Fonte Regio, 49, 90030 Castronovo di Sicilia (PA) and analysed as part of this study. All necessary permits were obtained for the described study from the Soprintendenza dei Beni culturali e ambientali di Palermo, which complied with all relevant regulations.

## Results and discussion

Using an acid methanol extraction procedure [28] (S1 Text), over 90% (122/134) of the ceramic samples yielded lipid concentrations above 5 μg g^-1^, with a mean concentration of 124.3 μg g^-1^ (CSP), 52.7 μg g^-1^ (PB), 96.7 μg g^-1^ (GA) and 154.56 μg g^-1^ (CLESP) respectively. A value of 5μg g^-1^ has previously been deemed the lowest lipid concentration that can be reliably attributed as endogenous and therefore interpretable [40, 41].

### Evidence of animal products

Animal products undoubtedly played an important part in cuisine at this time. Meat was considered a staple in Arabic- Islamic cuisine where the regular dish typically contained meat [42]. Lamb and mutton, chicken and dairy products (milk, yogurts and cheeses) appear regularly in accounts of cuisine, where mutton was considered a delicacy consumed by the upper classes [24–26]. The consumption of pork is forbidden as part of the Islamic religion, which is reflected by its absence from culinary literary sources. However, the complete absence of pork in Sicily during this time cannot be assumed. For all of the four sites investigated, faunal remains of caprine (both sheep and goats), cattle and domestic fowl have been identified [22, 23, 43] (S4 Text). Faunal analysis at PB and GA have shown the near absence of suid remains compared to a higher abundance at the rural site of CLESP [22, 23, 43]. In contrast to the other sites in Palermo, suid remains were identified at CSP [22, 23, 43] (S4 Text). The presence or absence of faunal remains in the archaeological record can, to some extent, inform us about how the meat or secondary products from these animals were processed through culling patterns, butchery and burning marks. Through organic residue analysis of ceramic cooking wares, we were interested in how animal products were incorporated into culinary practices at the site level.

**Fig 2:**
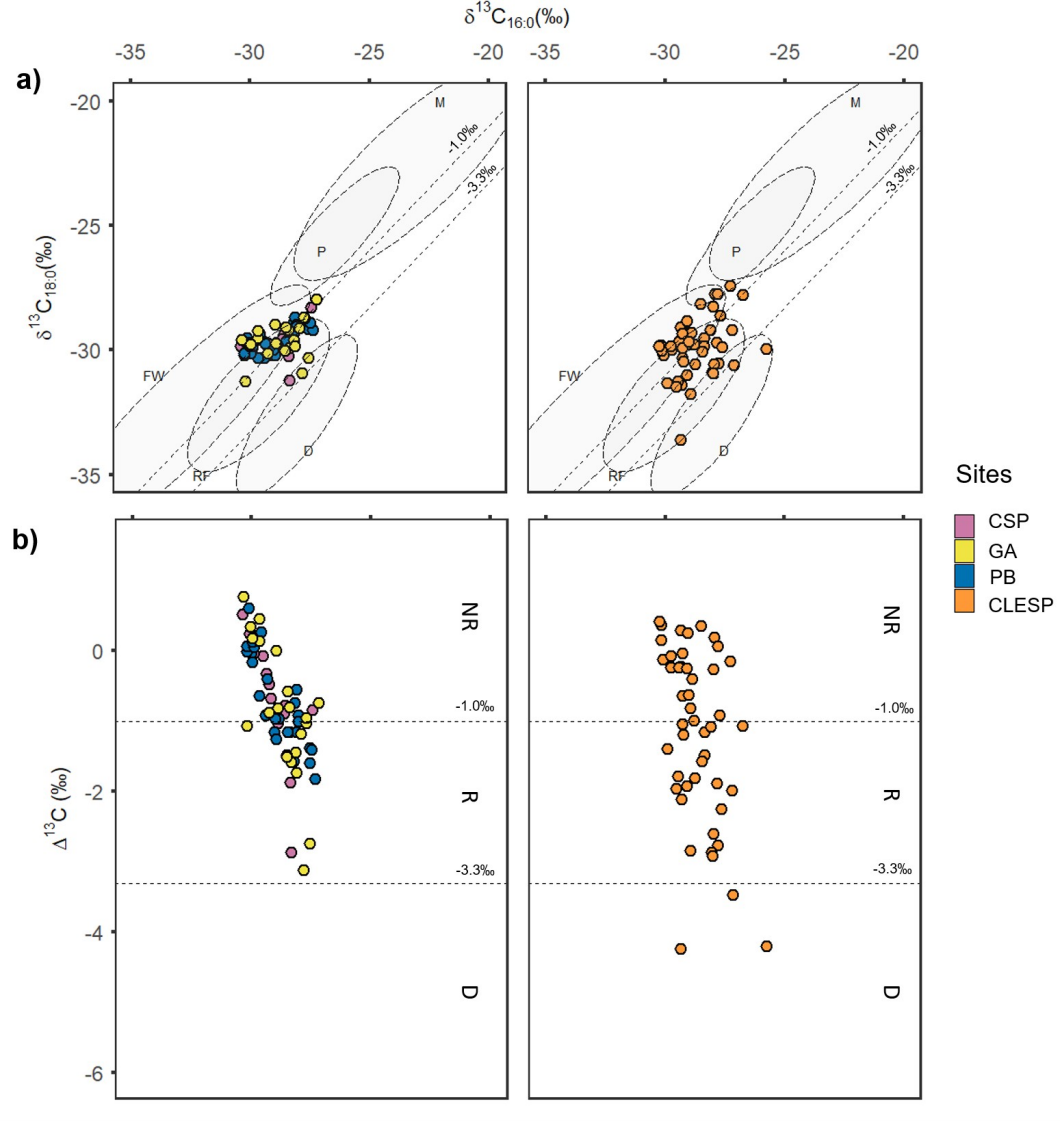
Plots of fatty acid stable isotope values obtained from individual vessels from Sicilian Islamic pottery. **a)** Plot of δ^13^C_16:0_ against δ^13^C_18:0_. Ranges (68% confidence) of 269 modern authentic reference products are shown, D (Ruminant dairy), RF (Ruminant adipose), P (Porcine), M (Marine), and FW (Fresh water). These references are published elsewhere [47] **b)** Plot of Δ^13^C against δ^13^C_16:0_. values <-3.3‰ are typically associated with D (Ruminant dairy), values between -3.3‰ and -1.0 ‰ are associated with R (Ruminant adipose) and above -1.0‰ can be considered as NR (Non- ruminant) [45].

Degraded animal fats were the most common lipid profiles encountered, characterised by an equal dominance of palmitic (C_16:0_) and stearic acids (C_18:0_) and the presence of cholesterol. Of these, a large proportion (90%) had branched and linear C_15:0_ and C_17:0_ fatty acids which are indicative of ruminant fats, formed through bacterial transformation of lipids in the rumen [44]. To further understand the origin of these lipids, 122 of the extracts were analysed by GC-C-IRMS to determine the stable carbon isotope value of the major fatty acids (C_16:0_ and C_18:0_) (S1 Text and S1 Data). This approach has shown to be useful for distinguishing ruminant adipose (i.e. carcass fats), non-ruminant adipose and ruminant dairy fats based on the difference Δ^13^C between the δ^13^C values of these fatty acids [30, 31, 45, 46] as well as marine and freshwater resources based on their absolute values [27] (Fig 2).

A high proportion of samples from all Palermo sites (64%) and CLESP (40%) fall within a relatively narrow range matching reference values of non-ruminant fats. However, no samples yielded fatty acid δ^13^C values that fall directly within the range of modern porcine fats which tend to have fatty acids more enriched in ^13^C than the values presented here (Fig 2A). At Palermo, these findings are in agreement with the near absence of suid remains at PB and GA [23, 43] (0.8% and 1.61% NISP respectively) (S4 Text). Although suid remains are in higher abundance at CSP [22] (14.7% NISP) (S4 Text), it may be suggested that pork was not selected and processed in the ceramic containers. It is interesting to note that four samples from CLESP are clustered towards more enriched δ^13^C values and fall on the edge of the range of the reference porcine fats (Fig 2A). The processing of pork in the ceramic containers at CLESP would agree with the faunal evidence at this site whereby domestic suids are the second most dominant species in the faunal assemblage at CLESP (32% NISP) [22] (S4 Text). However, the presence/absence of porcine fats based on these δ^13^C values must be treated with caution as the reference ellipses provided here for modern porcine from Northern Europe may not be representative of early medieval Sicilian porcine values due to the variability of δ^13^C _16:0_ values dependant on the animals diet [48].

Furthermore, the depleted δ^13^C _16:0_ values (~-30 ‰) within the range of non-ruminant fats from all sites are difficult to interpret. These values could represent the processing of other non-ruminant animal fats such as domestic fowl [49] or hare [50] which are represented in the faunal assemblages of these sites [22, 23, 43], but are present in very low proportions (>5% of the total NISP at all sites) (S4 Text). The contribution of C3 plant products such as vegetable oils and cereals etc., can impact δ^13^C values falling in the range of non-ruminant products. However, in most cases, the molecular profiles of these samples are dominated by C_16:0_ and C_18:0_ in equal proportions (Fig 4) and the presence of plant biomarkers is not consistent with samples that fall within the range of non-ruminant products. Thus, it is difficult to determine the source of these δ^13^C values and indeed mixtures of non-ruminant animal products, plant products and ruminant animals cannot be dismissed.

**Fig 3:**
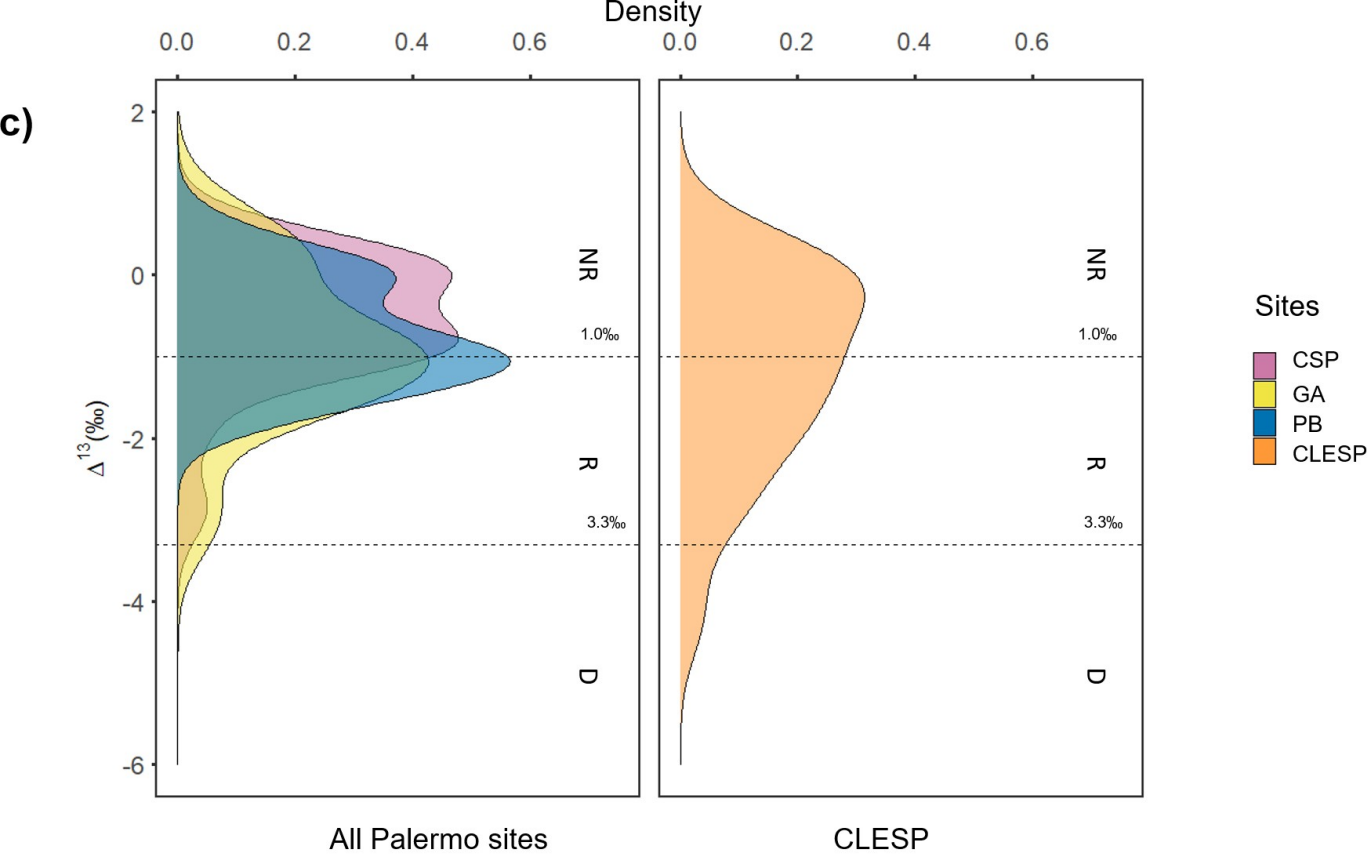
Kernel density estimate of Δ^13^C values. Bandwidth = 0.5. Δ^13^C values <-3.3‰ are typically associated with D (Ruminant dairy), values between -3.3‰ and -1.0 ‰ are associated with R (Ruminant adipose) and above -1.0‰ can be considered as NR (Non- ruminant) [45].

A number of fatty acids δ^13^C values obtained from the pottery from all sites fall within the range of ruminant adipose fat (CSP *n* = 3; GA *n* = 8; PB *n* = 9; CLESP *n* = 20) (Fig 2). The incorporation of ruminant animal products in these vessels is supported by the high dominance of ruminant species at these sites (cattle and caprines) [22, 23, 43] (S4 Text). It is important to note that the presence of ruminant fats may be underrepresented by the δ^13^C values, due to mixing of non-ruminant and ruminant fats. Of note, lipids from 3 samples from CLESP had δ^13^C values within the range of modern ruminant dairy products (Fig 2). This suggests that both primary and secondary ruminant products were processed in domestic containers at the rural site. Conversely, no evidence of dairy products was indicated by the fatty acid δ^13^C values obtained from the pottery from Palermo. Further use of dairy cannot be ruled out but here the signals are more difficult to interpret due to mixing between different types of animal fats and potentially plant oils, as shown by the density distribution of Δ^13^C values at this site (Figs 2B and 3). Interestingly, the Δ^13^C values from all the Palermo pottery are not normally distributed around the mean perhaps indicating distinct uses, where vessels are dedicated for specific roles (Fig 3). Whether these roles track specific food products or specific combinations of food products is more difficult to discern. In contrast, the Δ^13^C values from CLESP are much more widely dispersed compared to the samples from Palermo despite a similar sample size (Fig 3), perhaps indicating more generalised uses of pottery vessels.

### Further resolution with triacylglycerols (TAGs)

**Fig 4:**
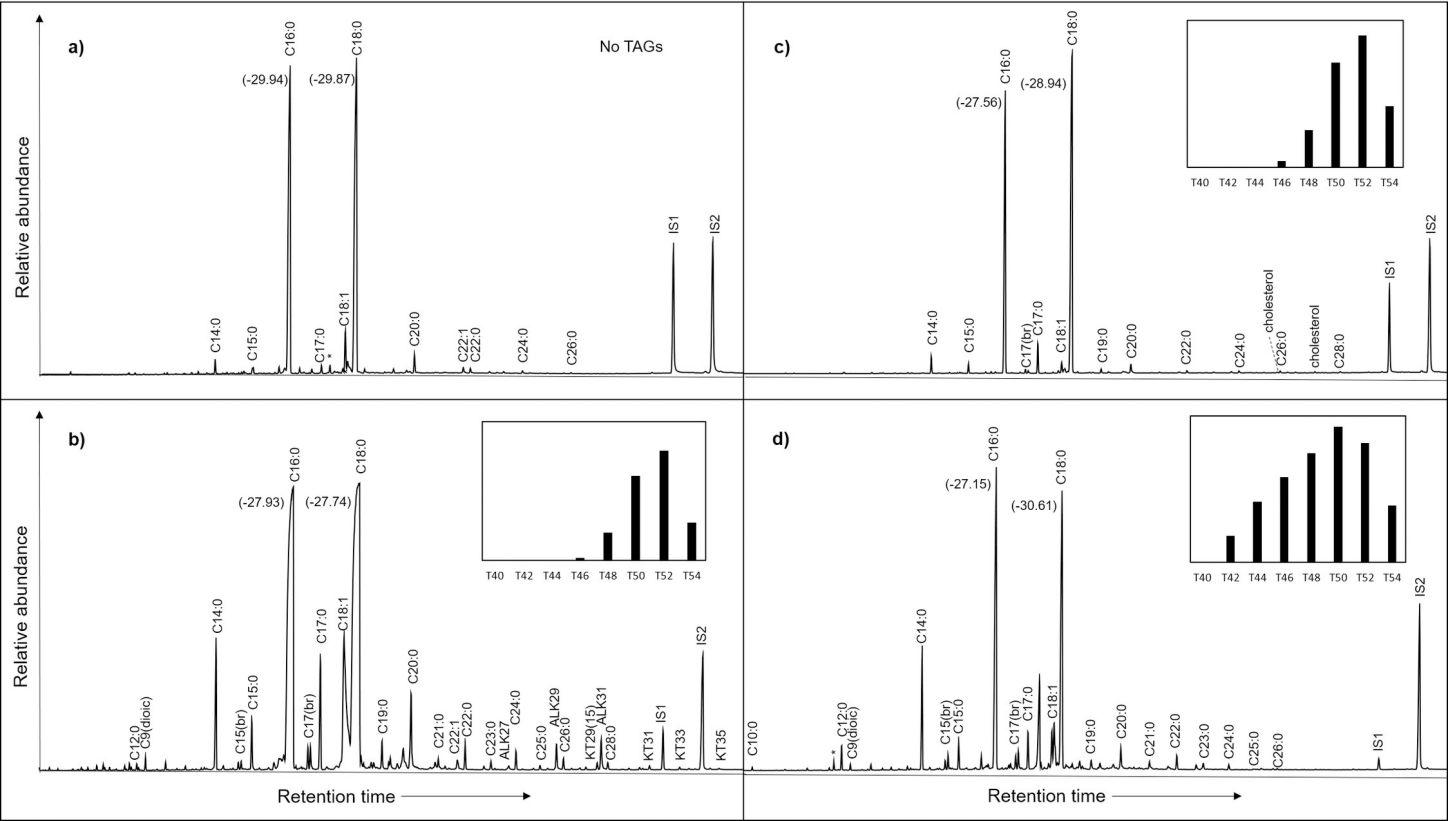
TIC chromatograms of pottery extracts. a) AE chromatogram of sample CSP_25 that yielded δ^13^C values within the range of non-ruminant products, b) AE chromatogram of sample CLESP_29 that yielded δ^13^C values within the range of non-ruminant products (porcine) and shows the TAG distribution profile associated with this sample after SE, c) AE chromatogram of sample GA_34 that yielded δ^13^C values within the range of ruminant products adipose products and shows the TAG distribution profile associated with this sample after SE, d) AE chromatogram of sample CLESP_26 that yielded δ^13^C values within the range of products and shows the TAG distribution profile associated with this sample after SE. Parentheses indicate the δ^13^C_16:0_ and δ^13^C_18:0_ fatty acid values.

The distribution and relative abundance of different triacylglycerols (TAGs), can help to further understand the origin of animal fats [31, 51]. Several samples extracted using solvent extraction (S1 and S2 Texts), yielded intact TAGs (32/106 samples). In most cases, the TAG profiles were characteristic of ruminant adipose fats (T_46_ to T_54_) (S2 Text). Unlike non-ruminant adipose fats, where there is a clear predominance of T_52_ over T_50_ and T_54_ and the distribution for ruminant fats is centred on T_52_ [31]. No samples yielded TAG profiles indicative of non-ruminant adipose fats and of note, 3/4 samples from CLESP that fell within the δ^13^C values of modern porcine fats did not yield intact TAGs and 1 sample yielded a TAG profile indicative of ruminant adipose fats (Fig 4C). Thus, highlighting the clear evidence of mixing of products and the difficulty in interpreting these values. A broader range of TAGs (T_42_ to T_54_) is typical of dairy fats [31] and was identified in 2 samples from CLESP (Fig 4D) (S2 Text).

**Fig 5:**
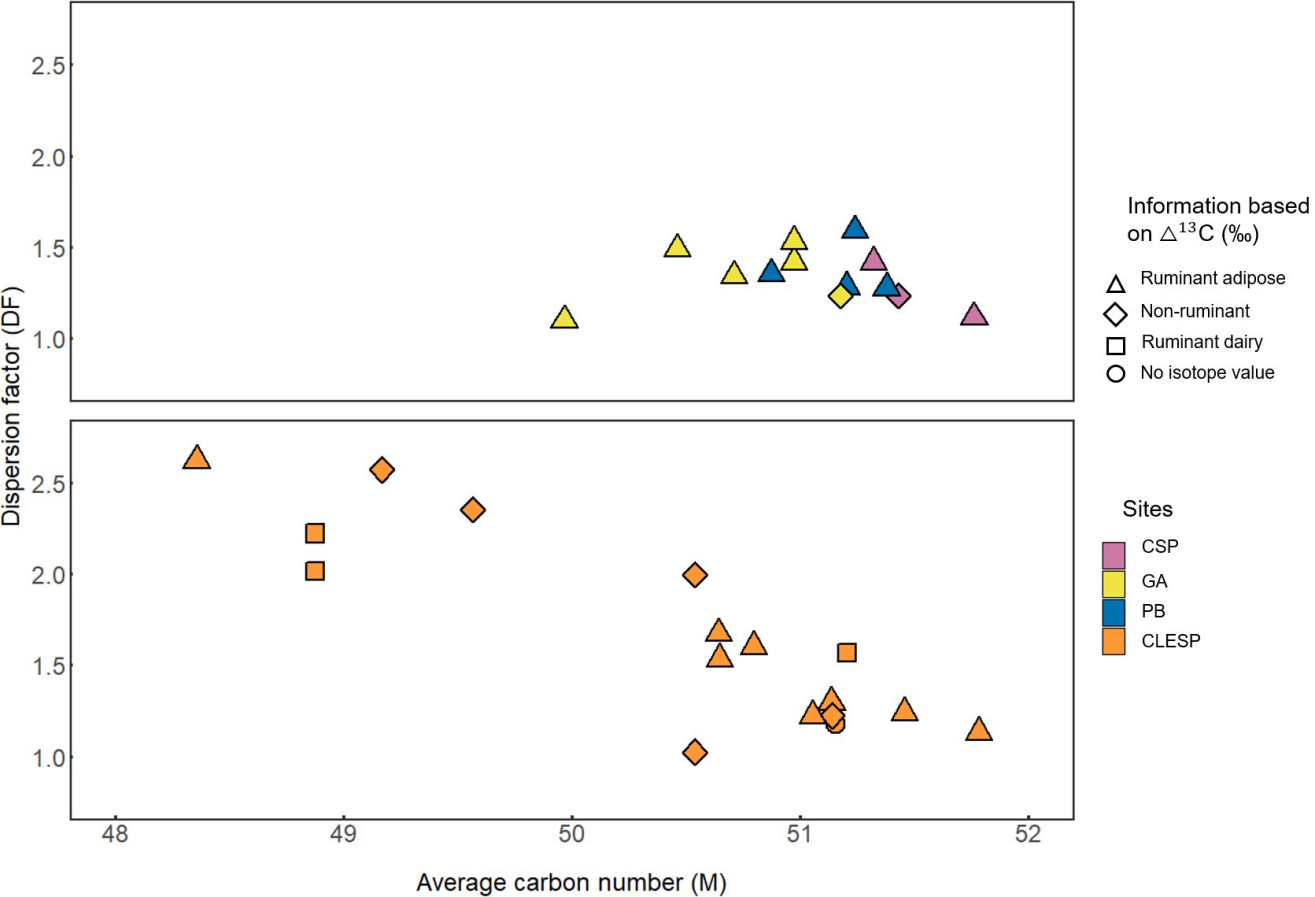
Plots of TAG information based on HT-GC data of individual pottery samples from Palermo sites (CSP, GA and PB) and Castronovo (CLESP) with interpretations based on fatty acid isotope values. The dispersion factor (DF) and average carbon number (M) were calculated using statistical equations outlined by Mirabaud et al. [52]. Shapes represent ruminant dairy (●) based on Δ^13^C values <-3.3‰, ruminant adipose (◆) based on Δ^13^C values between -3.3‰ and -1.0 ‰ and non-ruminant (■) based on Δ^13^C above -1.0‰ [45].

It has been shown that by plotting the average carbon number of the TAGs (M) and the dispersion factor (DF) ruminant adipose and dairy products can be broadly distinguished [52]. However, assigning specific products based on TAG profiles is undermined by preferential loss of lower molecular weight components [31] and mixing of resources. Nevertheless, samples from CLESP have a wider distribution of TAGs compared to those from Palermo (Fig 5) indicating the occurrence of dairy fats in five samples with TAGs preserved i.e., those with a low average carbon number (48–49) and a higher dispersion factor (2.0–2.6). In other cases, the TAG distributions more closely resemble ruminant carcass fats [52].

The correspondence between vessels categorised by their fatty acid stable carbon isotope values (ruminant, non-ruminant, and dairy) and the distribution of TAGs is not straightforward to interpret for CLESP samples (Fig 5). Samples plotting in the ruminant dairy isotope range would be expected to have a lower M value which is not always the case. Similarly, two vessels from this site have non-ruminant isotope values but a low M and high DF values, which is more typical of dairy. This suggests substantial mixing of products within the pottery in most cases, with perhaps two vessels used more exclusively for dairy. At the Palermo sites, there is little evidence for dairy by considering either the TAG distribution or their stable isotope values, evidence of a contrasting pattern of resource use between the urban centre and rural settlement. Animal husbandry orientated towards meat production has been observed at urban centres in Al-Andalus compared to rural settlements where a more mixed economy prevails [13, 14, 53].

### Evidence of aquatic products

The importance of fish and shellfish is not fully understood in Islamic Sicily. Fish is not generally considered in high regard in high-status Islamic cuisine and in Arabic recipes from the East fish is rare compared to meat, although fish recipes do appear marginally more frequently in al-Andalus [24] (p.191). When they are mentioned in recipes, both in Arab (Eastern) and Andalusian Medieval cookbooks, fish are baked or cooked in stews or sauces, rarely fried [54] (pp.264-265). A small number of tuna remains were identified at PB, GA and CSP as well the inland site of CLESP [22, 23, 43] (S4 Text). A bias must be considered in the recovery of fish remains from archaeological excavations, due to sampling biases and preservation. Tuna fishing is thought to have been abundant in coastal sites of Trapani and Palermo during this time and tuna trapping techniques were spread through the island by the Arabs after they arrived on the island [55, 56]. It may be assumed that at major coastal towns, such as Palermo, fresh fish were consumed and perhaps preserved (salted or dried, for the distribution of inland areas as observed in Roman sites inside and outside of the Mediterranean [57, 58].

Freshwater or marine organisms could not be identified in any of the vessels analysed based on their fatty acid δ^13^C values (Fig 2A). Similarly, specific lipids derived from heating freshwater or marine animals [30, 59, 60], were absent in all the vessels analysed despite the use of very sensitive approaches for their detection (S1 Text). Isoprenoid fatty acids that are at high abundance in aquatic oils were identified in a number of samples in all four sites (S1 Data), including phytanic acids and 4,8,12-trimethyltridecanoic acid (TMTD), although these are present in some animal fats, albeit at lower concentration. It was not possible to distinguish the source of phytanic acid further based on their stereoisomer ratios (%SSR), as has been suggested [61]. The %SRR of many samples fell within the range of both aquatic oils and ruminant fats (S1 Data). The absence of fish oils in ceramics from the coastal sites of Palermo is somewhat surprising given the presumed availability of fresh fish. However, human isotope evidence for fish consumption in other medieval coastal sites in the Mediterranean do not indicate a high consumption of marine products [62]. Although it is possible that fish were prepared and consumed in other ways (e.g. smoking, salting, cooked directly on fire, or processed as fish sauce) not detectable in the ceramics analysed here, these results likely reflect the lesser importance of fish in Arabic cuisine in contrast to other animal products [24] (p.191).

### Evidence of vegetables, fruits and cereals

In addition to meats and other animal products, vegetables, fruits and cereals likely played an important role in cuisine. With the Islamic green revolution, certain vegetables, fruits and cereals gained new importance and written sources of Islamic and complex mixtures of herbs, spices and vegetables are well documented in Arabic literature. Alongside spinach, aubergine and artichoke, other vegetables mentioned in historical sources include turnip, cabbage, cauliflower, onion, garlic and leek [42, 63] (pp.132-7). Furthermore, dishes often reflect a sweet and sour/ salty palate, where fruits and fruit juices were added to savoury meat dishes, for example citrus fruits (oranges and lemons), apples, pomegranates and grape products [42, 64]. Recent archaeobotanical evidence has identified several species of vegetables, fruits and cereals in Islamic contexts from Sicily including several species of plums (*Prunus* ssp.) at CLESP and citrus fruits, watermelon and aubergine at the site of Mazara del Vallo [8, 9]. However, in what way these resources were utilized in everyday cuisine is not fully understood.

In contrast to animal fats, the ability to assess the presence of plant products in archaeological ceramics is limited by their comparatively lower lipid yield [65]. Even when lipid profiles, indicative of a plant source, are encountered, they are rarely specific to a product/species. In order to maximise information, we have implemented an approach that involves the identification of leaf waxes, seed oils, phenolic lipids, terpenoids, fruit acids, cereal alkylresorcinols and miliacin. Lipid profiles with long-chain odd *n-*alkanes (with a predominance of C_29_ or C_31_), *n-*alcohols and wax esters (W_40_-W_48_) indicate plant waxes [66, 67]. When present, high oleic to stearic acid ratios (rare in archaeological samples) can indicate plant oils, alongside palmitic acid as a major constituent and sometimes unsaturated C_18:2_ [68, 69]. Short chain carboxylic acids (fumaric, succinic, malic, and tartaric) can be used to indicate the presence of fruit products [17, 29]. The presence of alkylresorcinols can be used to identify cereals (wheat, barley and rye) [70–72] and the presence broomcorn millet can be identified in archaeological ceramics by the presence miliacin (olean-18-en-3β-ol methyl ether) [73, 74].

Plant sterols (exclusively β-sitosterol) were identified in several samples across all four sites, often identified with other non-specific plant derived lipids. Spinach-specific sterols (α-spinasterol and 7-stigmastenol (S3 Text and S1 Data) were not identified in any vessels, possibly because these compounds are easily degraded when cooked in pottery [75]. *n*-alkane, *n*-alkanol and ketone distributions indicated the presence of plant waxes in several samples. Additionally, C_16:0_, C_18:0_, C_20:0_ and C_22:0_ wax esters were identified in a few samples, sometimes associated with odd-numbered *n-*alkanes, suggesting a plant wax or a mixture of plant wax and beeswax [67, 76].

**Fig 6:**
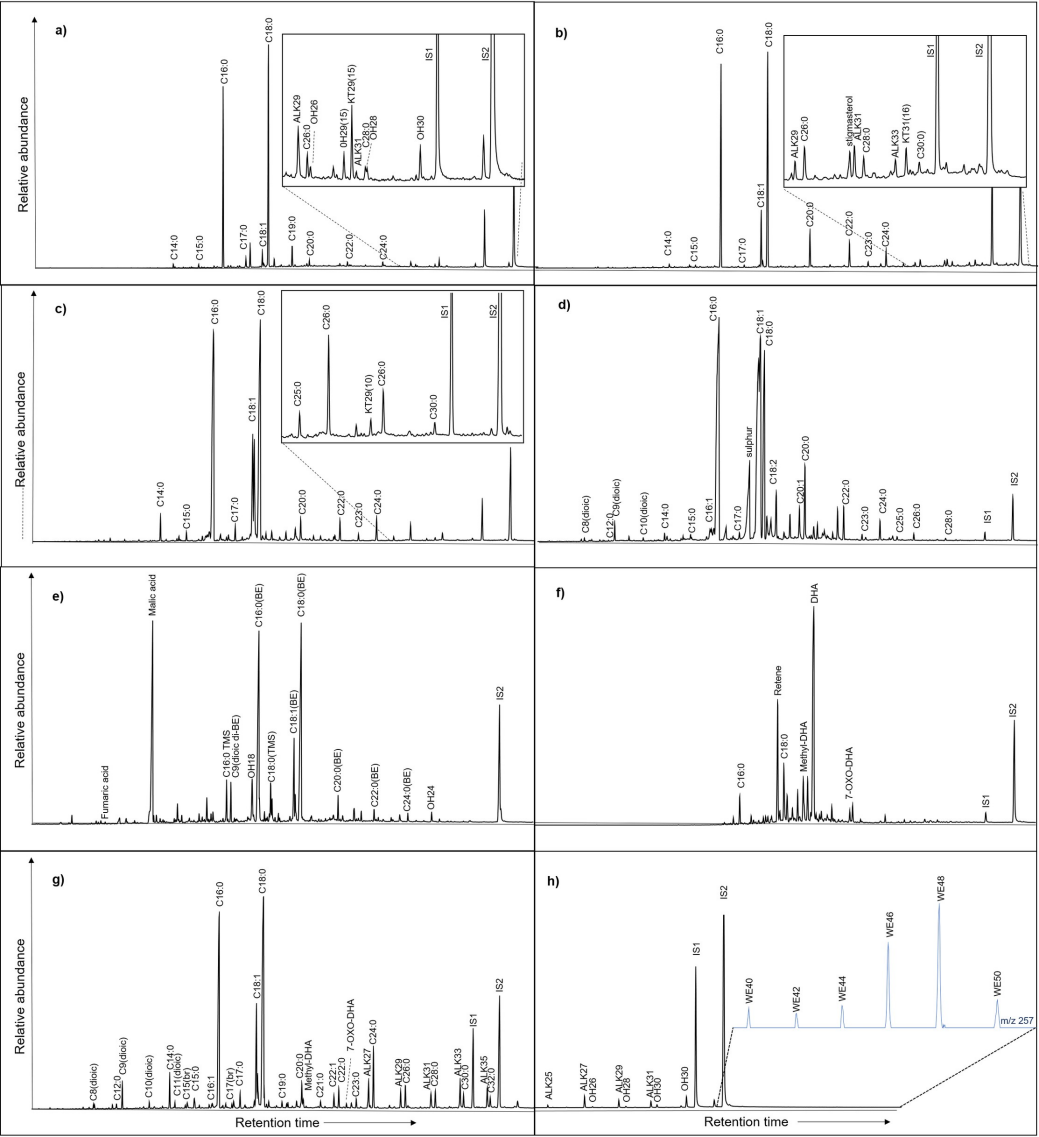
TIC chromatograms of extracts typical of a variety of products identified in these ceramics. a) AE chromatogram of sample GA_10 showing the presence of C_29(15)_ ketone that indicates the presumed presence of *Brassica* [9]. b) AE chromatogram of sample PB_26 showing the presence of C_31_ ketone (hentriacontane-16-one) that indicates the presumed presence of leek (*Allium porrum*) in the ceramic samples [10–13]. c) AE chromatogram of CLESP_58 showing the presence of C_29(10)_ ketone (nonacosane-10-one) that indicates the presumed presence of (*Foeniculum vulgare*) (Fennel) [14]. d) AE chromatogram of CLESP_12 indicating plant oil by a C_18:1_/C_18:0_ >2 and the presence of C_18:2_ [2, 15]. e) Chromatogram of sample CSP_2 showing the presence of malic acid after acid butylation. f) SE chromatogram of sample CSP_C5 indicating the presence of Pinaceae biomarkers: retene, methyl-dehydroabietic acid (Methyl-DHA), dehydroabietic acid (DHA), 7-oxo-dehydroabietic acid (7-oxo-DHA). g) AE chromatogram of sample CSP 4 typical of beeswax as well as animal fat and pine products. h) SE chromatogram of CSP 4 showing distribution of alkanes and alcohols typical of beeswax products alongside HT-GC of ion 257 showing the distribution of WE. Internal standards alkane C_34_ (IS1) and C_36_ (IS2) are shown.

In some cases, it was possible to offer greater taxonomic resolution regarding the origin of the leaf waxes as they display a specific *n*-alkane, *n*-alkanol and ketone distributions. Ketone specific for *Brassica* (nonacosane-15-one) and alcohol nonacosane-15-ol [77] alongside other degraded leaf waxes were found in several samples (*n* = 12) (Fig 6A). Hentriacontane-16-one (C_31_ ketone) and *n-*hentriacontane (C_31_ alkane) in one sample from PB could be attributed to leek [78–80] (Fig 6B). Additionally, nonacosane-10-one, the major ketone found in broad-leaved sermountain (*Laserpitium latifolium*; [81]) and fennel (*Foeniculum vulgare* [82]) was detected in pottery samples from CLESP (*n* = 5) (Fig 6C). Nonacosane-10-one may also be present in other apiaceous, for example *Apium* sp. However, the presence of nonacosane-10-one in archaeological ceramics has not previously been reported and no other biomarkers were detected to firmly identify any of these plants. Finally, one sample from CLESP indicated the presence of a plant oil, by a relatively high oleic to stearic acid ratio (C_18:1_/C_18:0_ >2) in addition to a small amount of linoleic acid (C_18:2_) [68, 69] (Fig 6D). Plant oils are likely underrepresented in these cooking vessels as oleic acid is susceptible to degradation in the burial environment and through prolonged cooking events [83] and mixtures with animal products is likely to mask their presence [84].

**Fig 7:**
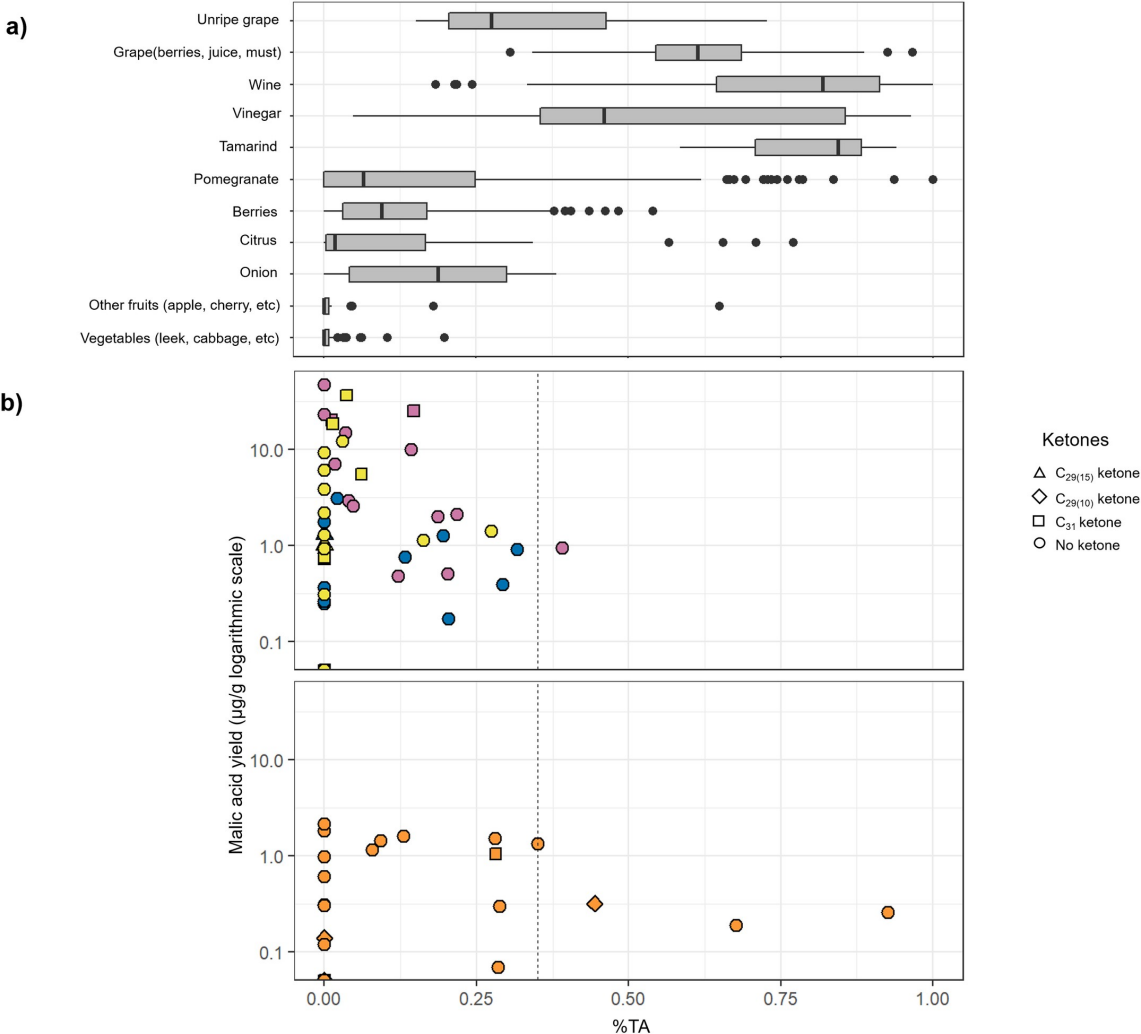
Malic acid yields and % tartaric acid (TA). a) Proportions of tartaric acid in various plants and plant products [17] (S1 Table); b) Proportions of tartaric acid in CLESP and Palermo cooking pots, plotted against the amount of malic acid extracted. % TA = tartaric acid/(tartaric + malic acid) [17]. C_29(10)_ ketone (nonacosane-10-one) indicates the presumed presence of broad-leaved sermountain (*Laserpitium latifolium*) [81] or (*Foeniculum vulgare*) (Fennel) [82]. C_29(15)_ ketone indicates the presumed presence of Brassica [77]. C_31_ ketone (hentriacontane-16-one) indicates the presumed presence of leek (*Allium porrum*) in the ceramic samples [78–80, 91].

Small organic acids, which are relatively insoluble in organic solvents were extracted from > 50% of the samples, using an acid butylation extraction developed by Garnier and Valamoti (2016) [17, 29, 85]. Malic and tartaric acids were identified, in variable amounts, in 97% and 70% of them, respectively. Succinic acid, which occurs in a variety of food products and can form through the degradation of fatty acids, was also present in a number of samples (59%) [81]. Fumaric, maleic, malonic, and oxalic acids were detected less frequently, in 10%, 14%, 8% and 3% of the samples respectively. Although tartaric acid is one of the main acids in grapes and wine, its mere presence is not sufficient to formally identify these products in CLESP and Palermo vessels, since it also exists in other plants [17, 85] and low amounts may be attributed to contamination from the burial environment [85]. Following the recommendations suggested by Drieu et al. (2020) and (2021), we performed a quantification of tartaric acid to consider only vessels with a significant amount of tartaric acid, which is unlikely to be contamination [17, 85]. Comparison of the proportions of malic and tartaric acids in the pots was used to distinguish between the presence of grapevine products and that of other plants (Fig 7) [17, 85–88] (S3 Text). Four vessels from CLESP and one sample from CSP produced a ratio of tartaric acid to the sum of tartaric and malic acids (%TA) of greater than 35%, characteristic of ripe grapes and their products (wine, juice, vinegar), as well as tamarind and some pomegranate cultivars [17, 85] (Fig 7B). Indeed, studies have suggested that cooking wares may have been used for the storage or heating of wine as well as being used as a flavouring for food in medieval Florence and Piombino [89, 90]. Interestingly, a long chain odd *n-*alkane distribution where alkane C_25_ is dominant, similar to the profile seen in the epicuticular wax of grape berries [66], was detected in a vessel that had the highest absolute amount (3.2 μg g^-1^) and relative % of tartaric acid (93%). The addition of whole fresh grapes and raisins to medieval Islamic dishes is mentioned in the literature [25].

Malic acid is one of the most common small acids in the plant kingdom and is present in large quantities in fruits [92]. In many samples of CSP and GA (Figs 6E and 7B), we can suggest the presence of fruits characterised by low proportions of tartaric acid compared to malic, such as apple, plum, cherry or peach (Fig 7A) [17, 86–88]. However, as malic acid is not restricted to fruit, it may have been derived from the other identified plant products, such as Brassicas or leeks [93].

Alkylresorcinols from cereals (wheat, barley or rye) were not found in any of the samples. However, as they are minor constituents and highly susceptible to degradation, their absence does not exclude cereals in these vessels [70–72]. Additionally, there was no evidence of broomcorn millet (*Panicum miliaceum*) in any of the vessels despite the fact that this product can be routinely identified through the presence of a specific biomarker (miliacin; olean-18-en-3β-ol methyl ether) [73, 74]. This supports preceding evidence from stable isotope analysis that millet was mostly consumed in the north of Italy during the Medieval period but not in the south [94]. Furthermore, no archaeobotanical evidence of millet has been identified in medieval Sicilian contexts.

### Resins and Beeswax

A residue typical of Pinaceae resin, including diterpenoids (abietic, primaric and isoprimaric acids) and their oxidation by-products (mainly dehydroabietic acid, 7-oxo-dehydroabietic and 15-hydroxy-dehydroabietic acid) were present in several of the Palermo vessels (5 CSP *n* = 5; GA *n* = 1; PB *n* = 4) [95, 96]. There are a number of examples in the archaeological record where Pinaceae resins have been used as a waterproofing agent for ceramics, mainly in amphorae to store liquids [97–99]. Due to the low melting point of pine resin, when used to line cooking vessels the pine may impact the flavour of the contents, a taste that was favoured in ancient Greece and Rome, but also today [100]. Retene and methyl dehydroabietic acid were present in one olla sample from CSP (Fig 6F). The presence of methyl dehydroabietic acid indicates that the resin has been heated with wood, likely through the production of pitch or tar [101, 102]. Pitch or pine tar may have been stored and prepared in the cooking pots for use as a sealant, adhesive or to waterproof boats as observed in other medieval contexts [89, 90, 103]. There are also accounts of dipping the stem of fruits in pitch (grapes and pears) to preserve them [42, 104]. Pears with red dipped stems are still present at Italian markets today [104].

Alongside evidence of pine products, one sample yielded a distribution of long-chain odd n-alkanes (C_25_-C_33_, predominance of alkane C_27_), long chain even FAs (C_20:0_-C_28:0_, predominance of FA C_24:0_), and alcohols (C_24_-C_32_, predominance of C_30_) and palmitic acid wax esters (W_40_-W_50_) indicative of beeswax products [105]. Additionally, this sample displayed evidence of animal and plant products (Fig 6G and 6H). A mixture of beeswax and pine resin has been observed before in archaeological ceramics from Neolithic, Roman and Medieval contexts for example in England, Egypt, France and Greece [106–108] as well as in Sicilian Bronze age ceramics [109], with the suggestion that it provides an effective way to waterproof or repair vessels [106–108]. However, the presence of beeswax in pottery may not be due to technological uses. Beeswax products have various uses, such as cosmetic, medicinal or can come from the presence of honey [108, 110]. The co-occurrence of other plant and animal products may indicate the use of honey as a sweetener, contributing to the sweetness of Islamic Arabic cuisine.

### Comparison of vessel forms and site variability

**Fig 8:**
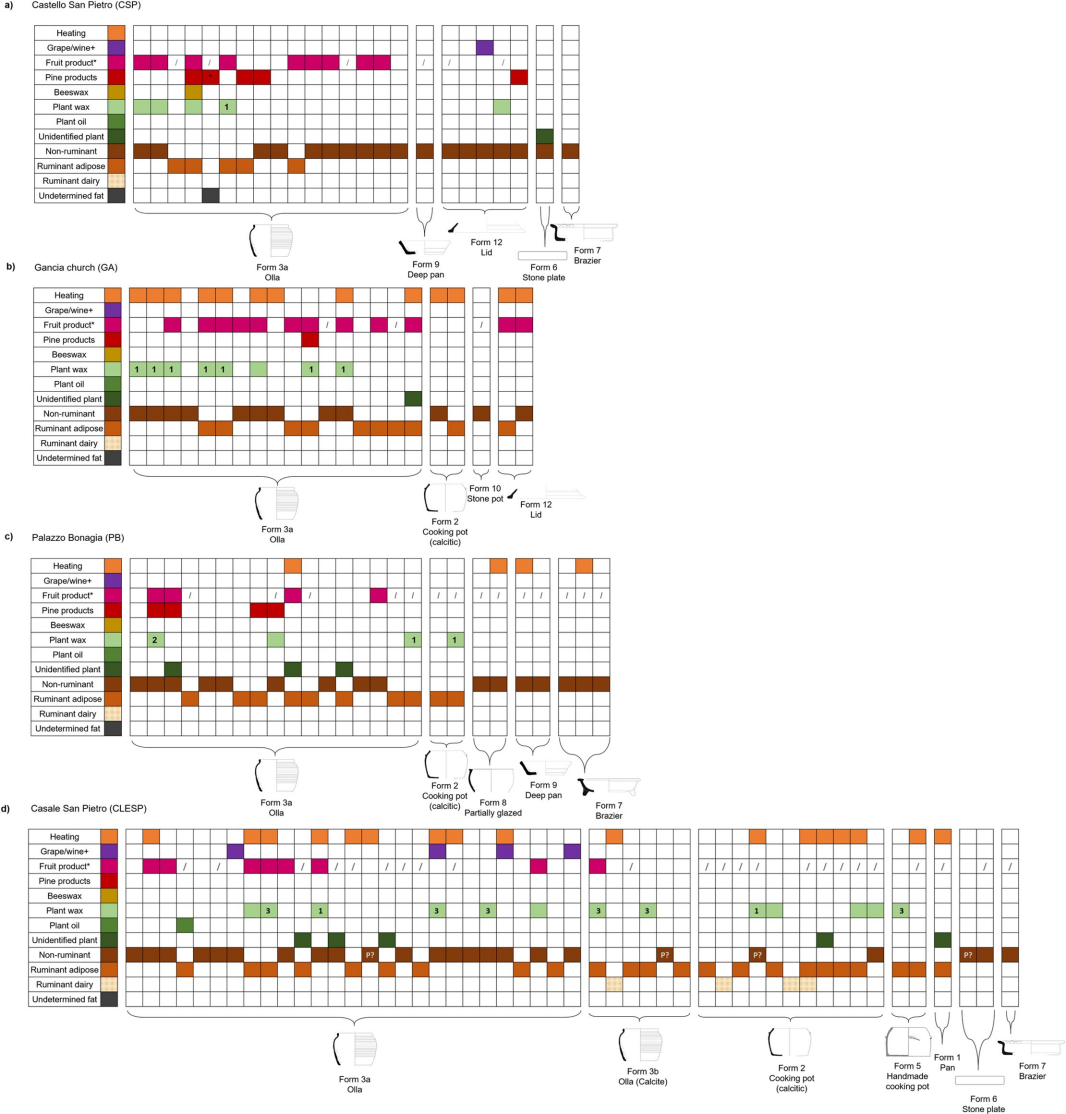
Summary figure of organic substances identified in pottery vessels from (a) CSP, (b) GA, (c) PB and (d) CLESP. Identification criteria of different commodities (ruminant dairy, ruminant adipose, non-ruminant, unidentified plant, plant oils, plant wax, beeswax, pine products, fruit products and grape products) are outlined in the text. Those not analysed for fruit acids are shown (/). Non-ruminant, ruminant and dairy were assigned based on Δ^13^C values. In one case both ruminant and dairy were identified based on clear dairy TAG distribution. Where P is noted in non-ruminant this refers to samples tentatively assigned to porcine. Specific taxonomy of plant waxes is indicated brassica (1), leek (2) and broadleaf sternum or fennel (3). In pine products pitch is indicated by (*). Evidence of heating was presumed in the presence of ketones C31, C33 and C35 [78] and/or APAAs [112, 113]. Vessel drawings used as examples based on actual vessels from CSP and PB [33, 34].

In Palermo generally, we found consistency in the use of cooking wares between sites with mixtures of animal fats, fruits and leafy vegetables without any clear distinction by vessel forms, such as between cooking pots and ollae (Fig 8A–8C). Although not detected in samples from CSP, thermal alteration molecular products were frequent in GA and PB and overall, the residue evidence suggests that ollae and cooking pots were general cooking wares, used to make stews or pottages. It is interesting to note that no clear difference in the use of ollae and cooking pots were observed in the sites of Palermo, despite differential techniques used to manufacture the two types of vessel [111]. The high frequency of fruit products in these general cooking wares, reflects notions about Islamic- Arabic cuisine, where fruits products are regularly documented as important accompaniments to salty meat dishes [24, 26, 42, 64]. Residues were also extracted from lids used to cover cooking vessels most likely during protracted boiling of the vessels. The presence of pine products, and in one sample beeswax products only found in ollae and lids, might suggest the importance of waterproofing these vessels to support more liquid dishes such as pottages and stews. Clear evidence of pitch identified in one olla sample from CSP may represent the reuse of these vessels for non-culinary uses such as the production or storage of pitch. It is interesting to note that pine products were not identified in samples from CLESP, either highlighting the differences in availability of conifer products or further supports the notion that pitch was being stored in these vessels and used to waterproof boats in the port of Palermo [89, 90]. Stone plates (form 6), braziers (form 7) and pans (form 1 and form 9) also contained animal products and plant products with little evidence of dedicated pottery use. Starchy cereals might also have been used but are difficult to detect using lipid residue analysis, despite a previous study, identifying starchy cereals in stone plates from Sicily [37].

In Casale San Pietro, we observed a similar consistency, with mixtures of animal fats, fruits and leafy vegetables present in all ceramic forms (Fig 8D). Of note, there was no evidence of waterproofing agents (pine products or beeswax) in samples from CLESP despite the fact ollae and cooking pots were seemingly used as general cooking wares for pottages and stews, as in Palermo. This may reflect a difference in the availability or need of these products between the urban centre and rural settlement. The presence of grape products/wine in four ollae samples from CLESP, alongside animal products and vegetable products suggests the integration of grape products such as wine or vinegar into cuisine at the rural site compared to limited evidence in samples from Palermo (1 sample from CSP).

Furthermore, evidence of porcine fats, tentatively identified in samples from CLESP, does not correlate with any particular vessel form (Fig 8D). The potential mixtures of pig fats, vegetables and fruits might suggest that their use was not tightly controlled, at least in a culinary sense. Conversely, in a number of samples from CLESP dairy is clearly separated from other products (i.e., animal adipose and plants). Additionally, in two samples where dairy products were present, thermal alteration markers were also identified. This suggests that vessels were dedicated for manipulation of dairy, perhaps in the production of ghee, yoghurts or cheeses. Of interest, evidence of dairy was only unambiguously identified in ‘calcite wares’ and not in other Palermo wares from CLESP or in samples from the Palermo sites. It cannot be assumed that dairy products were not being consumed at the Palermo sites and the lack of dairy traces in these vessels could be the result of the consumption of already processed dairy products (i.e., cheese) at these sites. However, we suggest this evidence constitutes not only a distinction between urban and rural resource use, and access to resources, but also sheds interesting light on specialised vessel use within the rural town. Although their provenance is still unknown, these wares were likely to have been imported, raising questions regarding the production and use of ‘calcite wares’ at CLESP as they are seemingly representative of local culinary practices.

During the early 9^th^ century, the inhabitants of Palermo were, at least to some degree, displaced by an incoming population and the city became increasingly urbanised. CLESP most likely benefited during the entire Islamic period being closely linked to the capital. The production of pottery at Palermo, and appearance of high-quality glazed wares at CLESP coincides with its rise in prosperity. However, it is likely that the capital also benefited from the rural site during this period as CLESP and possibly supplied the capital with resources such as grains and processed dairy products. Overall, the archaeological material and the contents of the vessels analysed in this study shows that, in this period (9^th^-12^th^ century), CLESP has an advantageous relationship with the capital, but there are differences in resource use at the site, most notably the production of dairy products in calcite ceramics, the integration of grape products in cuisine and possibly the consumption of pork. Future work on preceding periods should be undertaken to understand whether these differences reflect a differential impact of Islamic regimes at these sites.

## Conclusions

This study has provided the first direct evidence of cuisine from early medieval contexts in Sicily. We suggest that only by applying multifaceted organic residue analysis to many ceramic samples can complex cuisines begin to be untangled. The application of a range of analytical techniques and extraction methods enabled a wide variety of commodities and complex mixtures to be identified. For example, analytical procedures for the identification of small organic acids provided crucial evidence that fruits were incorporated in everyday culinary practices. A multifaceted analytical approach, including the identification of fruit products in domestic containers, has had its beginning in small corpus analysis of medieval ceramics [89, 90, 103, 114]. This research has for the first time applied these methods systematically to a large corpus of samples. Due to the lack of food crusts on the surface of the ceramics, an important caveat is that we are unable to distinguish whether foods were processed together to create complex ‘dishes’ or whether the residues build up over time [115] and therefore should be interpreted as a palimpsest reflecting multiple cooking events; indeed, both scenarios are likely.

Our main results can be summarised as follows:

1. Mixtures of commodities identified chemically, at all four sites across ceramic forms, are generally consistent with the colourful dishes noted in Arabic culinary literature, where meats, vegetables, and often fruits make up complex sweet, sour and salty recipes [24, 26, 42, 64]. The organic residue data certainly does not contradict the uptake of these culinary practices in rural and urban settlements across North West Sicily, as has been suggested [1, 2].
2. Terrestrial animal products were widely processed in ceramics from all four sites but there was no evidence of marine or freshwater products. This study supports evidence that fish may not have been considered as important as meat products in medieval Islamic cuisine although fish may have been prepared using non- ceramic techniques.
3. Dairy products were unambiguously identified at CLESP but were only present in calcite wares. This find depicts a preference of these specific wares for the manipulation of dairy products. The absence of dairy products in ceramics from the Palermo sites and their absence in other Palermo production wares in CLESP suggests a distinction in the use of pottery of different productions and gives some insights into exogenous and local food practices associated with these vessels.
4. Dedicated uses of other forms (stone plates, braziers and pans etc.) could not be identified.
5. Porcine fats were tentatively identified in pottery from the rural site of Casale San Pietro. Alongside evidence from the faunal assemblage at this site, this may reflect less stringent food taboos applied in rural areas or the presence of Christian communities as suggested in other studies [13, 14].
6. Brassicas, leeks and, possibly, fennel were identified providing an important complement to the archaeobotanical evidence. Fennel was exclusively found at the site of CLESP, where it grows prolifically today. However, specific biomarkers for other vegetable products (spinach, aubergine etc.) could not be identified.
7. The specific identification of grape products, more frequently in CLESP has opened questions regarding the use of these products and their integration into cuisine.
8. Non- culinary uses were identified in samples from Palermo sites in the form of pine products and beeswax products. This has provided insight not only into cuisine, but also pottery technologies during this period.

The evidence provided here should provide a useful baseline for further investigations aimed at examining continuity or change in pottery use as Sicily experienced profound social transformations during the Middle Ages, when under the control of different political powers. Furthermore, the analytical approach applied should provide a useful example to future studies of cuisine, particularly in contexts where complex mixtures of commodities might be expected.

## Supporting information

S1 Text Organic residue analysis methods. (DOCX)

S2 Text Triacylglycerol (TAG) distribution profiles. (DOCX)

S3 Text Organic residue analysis of modern plant products. (DOCX)

S4 Text Summary of faunal remains. (DOCX)

S1 Data Ceramic samples and organic residue analysis results. (XLSX)

S1 Table Malic acid and tartaric acid quantities in vegetables. (DOCX)
