# Supplementary material for: New insights into early medieval Islamic cuisine: Organic residue analysis of pottery from rural and urban Sicily
Source: PLoS One. 2021 Jun 9;16(6):e0252225. doi: 10.1371/journal.pone.0252225 (PMC8189454; doi:10.1371/journal.pone.0252225)
Supplement: S1 Text — (DOCX) [file pone.0252225.s001.docx]

**S1 Text. Organic residue analysis methods**

# Sampling of the ceramic

The ceramic sample was first ‘cleaned’ by removing the outer layer of the ceramic surface (approx. 1 mm deep) using a low speed Derma drill with an abrasive metal drill bit. The first millimetre of samples was discarded to avoid exogenous contamination. Approximately 2g of ceramic powder was obtained overall for analysis by drilling into the pot and collecting the powder.

# Extractions

## Acidified methanol extraction

Acidified methanol extraction method [[1]](https://paperpile.com/c/heOg87/fIxnb) is now the most commonly used method in archaeological samples that favour both high extraction yield and direct methylation for stable carbon isotopes measurements. The details of this extraction are recorded elsewhere [[1]](https://paperpile.com/c/heOg87/fIxnb) but in brief, 4 ml of methanol and 10 µl of alkane C_34:0_ were added to 1g of powdered ceramic sample and ultrasonicated. Sulphuric acid (H₂SO₄) was added to each sample and heated at 70℃ for 4 hours. After centrifuging, the supernatant was extracted and transferred to a clean labelled hatch tube. The samples were dried to completion under a gentle stream of nitrogen. The sample was then resuspended in hexane and 10 µl of alkane C_36:0_ was added as an internal standard before further analysis by gas chromatography techniques [[2]](https://paperpile.com/c/heOg87/DdPUQ).

## Solvent (SE) / total lipid extraction (TLE)

Solvent extraction (SE) method [[3]](https://paperpile.com/c/heOg87/Mbcp7) ensures the preservation of the most-informative molecules in fatty and waxy products (triacylglycerols and wax esters). It is worth noting that these are not always preserved in archaeological samples. The preparation for these samples was the same as with acid extraction and 1g of sample powder was used. After the addition of 10 µL of *n*-alkane C_34:0_, 5ml of DCM: MeOH (2:1, v/v) was added to each ceramic sample including the blank and standard. The samples were then sonicated for 15 minutes. They were then centrifuged for 10 mins. The supernatant was then transferred to a clean labelled hatch tube. These steps were performed 3 times. The solution was then reduced to 1-2 ml under a gentle stream of nitrogen and transferred to a large hydrolysis vial. This was then dried to completion under a gentle stream of nitrogen.

## Acid butylation extraction

Small organic acids were extracted following the acid butylation extraction protocol developed by Garnier and Valamoti (2016) [[4]](https://paperpile.com/c/heOg87/etFYN). In brief a BF 3 -butanol/hexane mixture (1:2,v/v) was added to the remaining powder previously extracted using the SE methods described above. The mixture was then heated for 2 hours at 80°C. After centrifuging, the supernatant was transferred to a clean vial and neutralised using a saturated sodium carbonate solution. The samples were extracted in DCM 3 times, and washed twice with distilled water. The extracts were then dried to completion under a gentle stream of nitrogen.

# Trimethylsilylation (TMS)

The dried extracts from solvent, acid butylation extraction and some select acidified methanol extracts were derivatized using *N, O*-bis(trimethylsilyl) trifluoroacetamide (BSTFA) with 1% trimethyl-chlorosilane and heated for 1 hour at 70℃. The solution and excess BSTFA was dried to completion under a gentle stream of nitrogen. 90 µl of *n-*hexane was added to re-dissolve the extract and vortexed before transferring to an auto sampling vial which contained 10 µl of the second internal standard C_36:0._

# Instrumentation

Gas chromatography - Flame ionisation detector (GC-FID) was used to screen for fatty acids and for basic quantification (methyl esters and TMS derivatives). Gas chromatography-Mass spectrometry (GC-MS) was used to identify complex molecules according to their mass and their fragmentation pattern.

## Gas chromatography - Flame ionisation detector (GC-FID)

Acidified methanol extracts were screened by gas chromatography fitted with a flame ionisation detector (FID) for quantification and general screening of preservation. An Agilent 7890A Series gas chromatograph was used fitted with a DB1-High temperature (HT) column (15 m × 0.32 mm × 0.1 μm). 1 µl of sample was injected via a splitless injector maintained at a temperature of 300°C. The temperature of the column was kept at 100 °C for 2 minutes and then increased by 20 °C every minute until a final temperature of 325 °C was reached. 325 °C was then held for 2 mins. Helium was used as the carrier gas at constant flow. The detector was kept at 300 °C with hydrogen flow of 30 ml min ^1^. This was a short temperature programme for 20 minutes.

TMS solvent extracts were screened with the same GC-FID instrument, fitted with the same HT column. The temperature programme was adapted to detect the presence of TAGs and wax esters and for quantification.  The temperature of the column was kept at 50 °C for 2 minutes and then increased by 10 °C every minute until a final temperature of 375 °C was reached. A temperature of 375 °C was then held for 10 mins. This was a longer temperature programme for 44.5 minutes.

## Gas chromatography - Mass spectrometry (GC-MS)

All extracts were analysed using gas chromatography mass spectrometry to identify biomarkers. The GC component was an Agilent 7890A series chromatography attached to an MS Agilent 5975 Inert XL mass selective detector with a quadrupole mass analyser (Agilent technologies, Cheadle Cheshire, UK). A DB-5MS (5%-phenyl)- methylpolysiloxane column (30 m x 0.250 mm x 0.25 µm; J&W Scientific, Folsom, CA, USA) was used. The GC column was inserted directly into the ion source of the mass spectrometer. 1 µl of sample was injected via a splitless injector maintained at a temperature of 300 °C. Helium at constant flow was used as the carrier gas. The ionisation energy of the spectrometer was 70eV and spectra were obtained by scanning between *m/z* 50 and 800. The temperature of the column was kept at 50 °C for 2 minutes and then increased by 10 °C every minute until a final temperature of 325 °C was reached. 325 °C was then held for 15 mins. Thus, the total run time was 44.5 minutes. Compounds were identified using Agilent Chemstation software with the NIST 14.0 mass spectral library.

## Gas chromatography - Mass spectrometry (GC-MS) for aquatic biomarkers (DB23 AQUA SIM)

Predominantly with the aim to search for *ω*-(*o*-alkylphenyl) alkanoic acids (APAAs) which can be formed through the heating of either plant or aquatic sources. Acidified methanol extracts were analysed on the same GC-MS instrument using a DB-23 ((50%-cyanopropyl)-methylpolysiloxane (60 m × 0.25 mm × 0.25 μm; J&W Scientific, Folsom, CA, USA) column. The temperature of the column was kept at 50 °C for 2 minutes and then increased by 10 °C every minute until 100 °C. The temperature increased then until 140 °C by 4 °C every minute, then until 160 °C by 0.5 °C every minute and finally until 250 °C by 20 °C every minute. To target ions of APAAs and other aquatic markers such as 4,8,12-trimethyl tridecanoic acid (TMTD), pristanic acid and phytanic acid, selected ion monitoring mode (SIM) was used. These were analysed using ChemStation software or Mass Hunter.

## High temperature HT-GCMS

TMS solvent extracts were analysed using a high temperature column and programme which was adapted to detect the presence of TAGs and wax esters. An Agilent 7890A Series gas chromatograph was used fitted with a DB5-HT column (30 m × 0.25 mm × 0.1 μm) instead of a DB-5MS and the temperature of the column was kept at 50 degrees for 2 minutes and then increased by 10 degrees every minute until a final temperature of 375°C was reached. The maximum temperature of 375°C was then held for 10 mins.  The total run time was 44.5mins.

## Gas Chromatography‐Combustion‐Isotope Ratio Mass Spectrometry (GC-C-IRMS)

Stable carbon isotope (δ^13^C) values of the major saturated fatty acids (FA; C_16:0_ and C_18:0_) were analysed by GC-C-IRMS. An Isoprime 100 (Isoprime, Cheadle, UK) with a Hewlett Packard 7890B series GC (Agilent Technologies, Santa Clara, CA, USA) and an Isoprime GC5 interface (Isoprime Cheadle, UK) was used. This was fitted with a DB-5MS ultra- inert fused silica column (US, 60m x 0.25mm x 0.25 µm). 1 µl of the sample was injected via a splitless injector maintained at a temperature of 300 °C. Helium 6.0 was used as the carrier gas at a constant flow rate of 3 ml/min. An Agilent 5975C mass spectrometer detector (MSD) was attached to the column and half of the gas eluting from the column was directed to, and ionized in the mass spectrometer. The other half of the gas eluting from the column was directed to the creature tube to oxidise carbon species in CO_2_.  The ionisation energy of the mass spectrometer was 70 eV and ion intensities of *m/z* 44, 45 and 46 were recorded.

IonOS software was used to compute the ^13^C/^12^C ratio of the peaks in the extracts. The ^13^C/^12^C ratio was calculated in comparison with a standard reference gas (CO_2_) of known isotopic composition. All samples were injected twice and only regarded if the mean standard deviation of both C_16:0_ and C_18:0_ was <0.3 ‰.  The △C values were expressed as per mill (‰) relative to the internal standard V-PDB (Vienna Pee Dee Belemnite) [[5,6]](https://paperpile.com/c/heOg87/TAm2K+ODDrn). This expression is defined by the following equation (equation 1) where R = : $\frac{\delta^{12}C}{\delta^{13}C}$

**
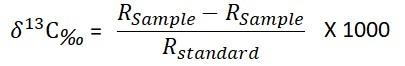
(Equation 1)**

An *n-*alkanoic acid ester standard of known isotopic composition (F8-3) was used to determine the precision and accuracy of the instrument which needed to remain <0.5 ‰ and <0.3 ‰ respectively. Values needed to be corrected to account for subsequent methylation of carboxyl groups during the acidified methanol extraction. These values were corrected in relation to the method standard (STD METHYL-n) a mixture of C_16:0_ and C_18:0_ FAs of known isotopic composition. This is shown by the following balance equation (equation 2):

**
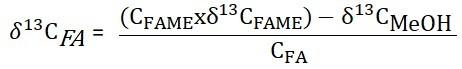
(Equation 2)**

# References

1. [Craig OE, Saul H, Lucquin A, Nishida Y, Taché K, Clarke L, et al. Earliest evidence for the use of pottery. Nature. 2013;496: 351. doi:](http://paperpile.com/b/heOg87/fIxnb)[10.1038/nature12109](http://dx.doi.org/10.1038/nature12109)

2. [Correa-Ascencio M, Evershed RP. High throughput screening of organic residues in archaeological potsherds using direct acidified methanol extraction. Analytical Methods. 2014;6: 1330–1340. doi:](http://paperpile.com/b/heOg87/DdPUQ)[10.1039/C3AY41678J](http://dx.doi.org/10.1039/C3AY41678J)

3. [Charters S, Evershed RP, Goad LJ, Leyden A, Blinkhorn PW, Denham V. Quantification and distribution of lipid in archaeological ceramics: implications for sampling potsherds for organic residue analysis and the classification of vessel use. Archaeometry. 1993;35: 211–223. doi:](http://paperpile.com/b/heOg87/Mbcp7)[10.1111/j.1475-4754.1993.tb01036.x](http://dx.doi.org/10.1111/j.1475-4754.1993.tb01036.x)

4. [Garnier N, Valamoti SM. Prehistoric wine-making at Dikili Tash (Northern Greece): Integrating residue analysis and archaeobotany. J Archaeol Sci. 2016;74: 195–206. doi:](http://paperpile.com/b/heOg87/etFYN)[10.1016/j.jas.2016.03.003](http://dx.doi.org/10.1016/j.jas.2016.03.003)

5. [Rieley G. Derivatization of organic compounds prior to gas chromatographic–combustion–isotope ratio mass spectrometric analysis: identification of isotope fractionation processes. Analyst. 1994;119: 915–919. doi:](http://paperpile.com/b/heOg87/TAm2K)[10.1039/AN9941900915](http://dx.doi.org/10.1039/AN9941900915)

6. [Woodbury SE, Evershed RP, Rossell JB, Griffith RE, Farnell P. Detection of Vegetable Oil Adulteration Using Gas Chromatography Combustion/Isotope Ratio Mass Spectrometry. Analytical Chemistry. 1995;67: 2685–2690. doi:](http://paperpile.com/b/heOg87/ODDrn)[10.1021/ac00111a029](http://dx.doi.org/10.1021/ac00111a029)
