# Supplementary figures and images for: New insights into early medieval Islamic cuisine: Organic residue analysis of pottery from rural and urban Sicily
Source: PLoS One. 2021 Jun 9;16(6):e0252225. doi: 10.1371/journal.pone.0252225 (PMC8189454; doi:10.1371/journal.pone.0252225)

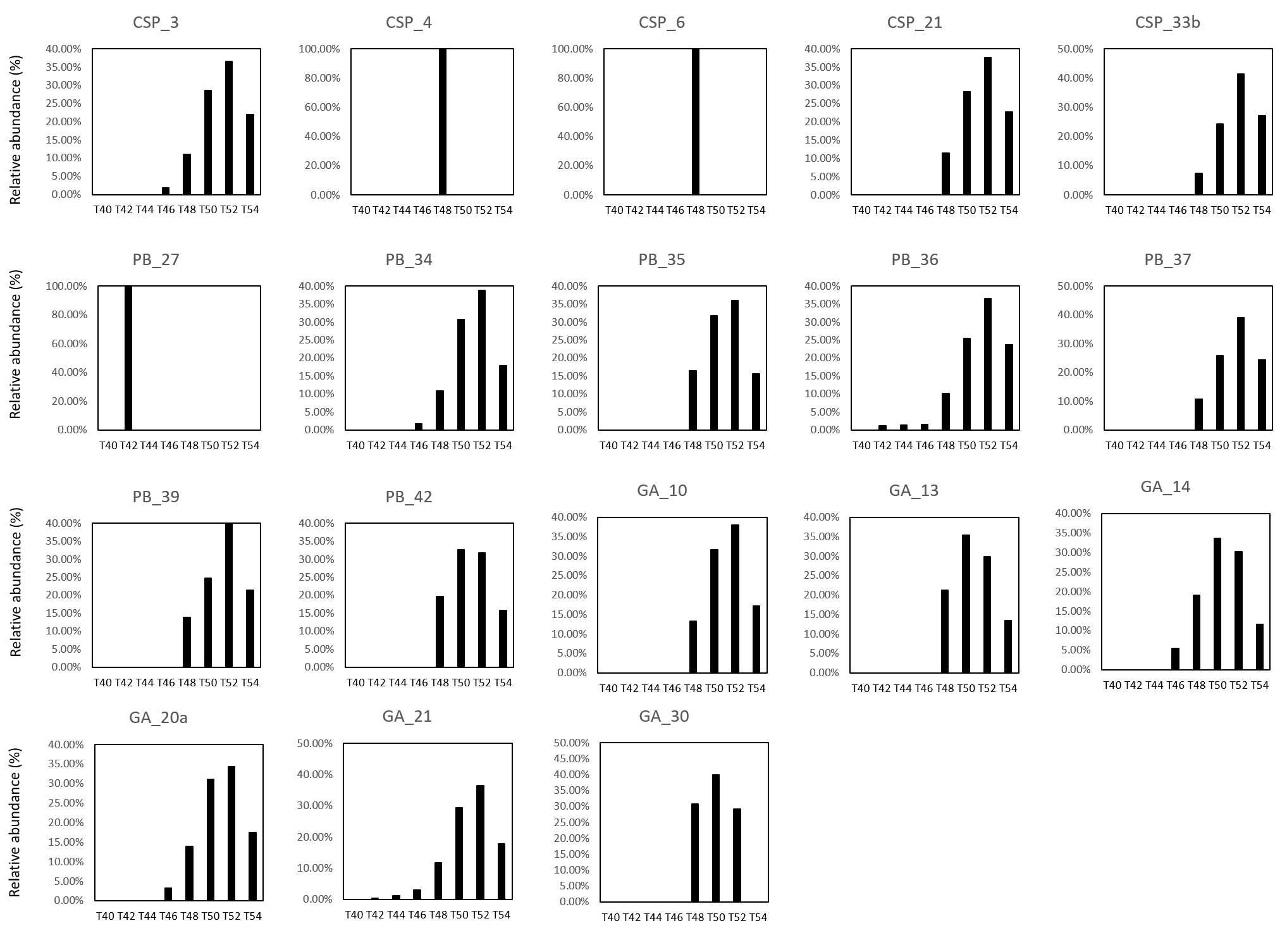


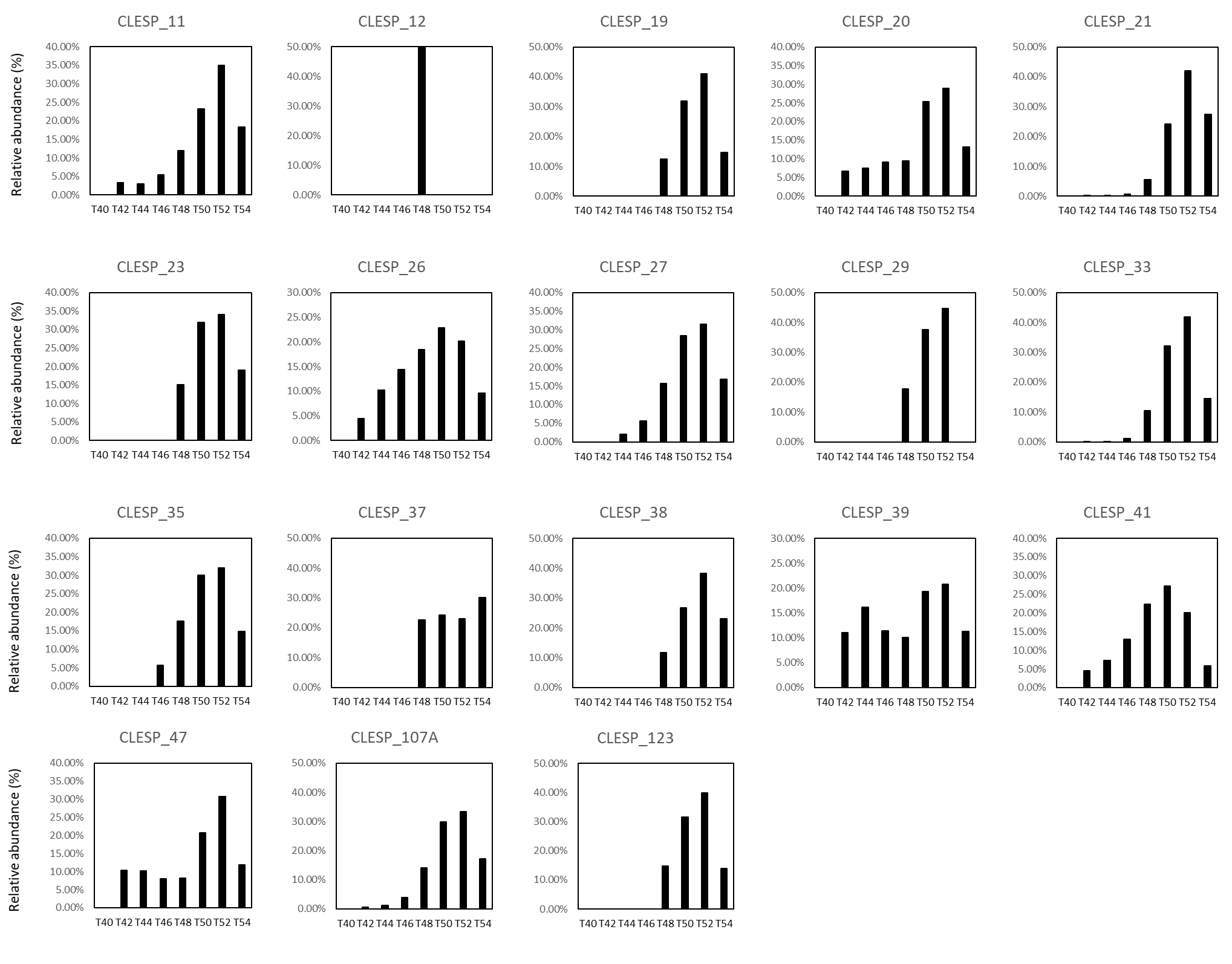

Supplement: S2 Text — (DOCX) [file pone.0252225.s002.docx]
