# Supplementary material for: New insights into early medieval Islamic cuisine: Organic residue analysis of pottery from rural and urban Sicily
Source: PLoS One. 2021 Jun 9;16(6):e0252225. doi: 10.1371/journal.pone.0252225 (PMC8189454; doi:10.1371/journal.pone.0252225)
Supplement: S3 Text — (DOCX) [file pone.0252225.s003.docx]

**S3 Text. Organic residue analysis of modern vegetables**

# **Introduction**

Different modern plants were extracted and analysed using the same well-established methods in organic residue analysis as the archaeological ceramic samples [[1–5]](https://paperpile.com/c/bQ9QdB/YFwLM+v5ooG+IKjcW+ShzKf+kaI4Z) (S1 text.). The aim was to identify possible biomarkers or characteristic molecular profiles to understand how these commodities may have been processed in the ceramic vessels. Here we analysed cabbage, leek, onion and carrot), as well as aubergine and spinach (thought to have appeared/developed in Sicily as part of the ‘Islamic green revolution’ [[6,7]](https://paperpile.com/c/bQ9QdB/Bb7lu+Ajxax). The samples were bought at the local market in Castronovo, Sicily or at the local supermarket in the UK (Table 1).

**Table 1: Modern vegetable samples analysed using ORA**. The table shows the type of samples and where they were acquired from.

| **Sample name** | **English** | **Acquired** | **Origin** |
| --- | --- | --- | --- |
| CA_MOD_1 | Turns (It: Giri di Campania) | Local market in Castronovo | Castronovo |
| CA_MOD_2 | Cabbage ( It: Cavoli) | Local market in Castronovo | Castronovo |
| CA_MOD_5a | Carrots leaves | Local market in Castronovo | Castronovo |
| CA_MOD_5b | Carrots | Local market in Castronovo | Castronovo |
| CA_MOD_7 | Aubergines | Local market in Castronovo | Castronovo |
| CA_MOD_16 | Leeks | UK supermarket | UK |
| CA_MOD_17 | Onion | Local market in Castronovo | Castronovo |
| CA_MOD_18 | Cabbage | UK supermarket | UK |
| CA_MOD_19 | Kale | UK supermarket | UK |
| CA_MOD_20 | Spinach | UK supermarket | Italy |

## **Results**

The samples delivered mainly saturated (C_16:0_, C_18:0_) and unsaturated (C_18:1_, C_18:2_, C_18:3_) fatty acids, sugars and sterols. Since sugars and unsaturated fatty acids are rapidly degraded, they were not considered for comparison with the archaeological samples. β-sitosterol is present in all samples except spinach. Spinach yielded a specific sterol signature composed of α-spinasterol and 7-stigmastenol, alongside *n-*alkane C_31_ and *n*-alcohols C_22_-C_26_, where C_26_ is dominant. In addition to β-sitosterol, aubergine and carrots delivered only stigmasterol, and onions only cycloartenol. Both stigmasterol and cycloartenol are not distinctive sterols and therefore cannot be used as a specific biomarker for these vegetables.

Cabbage revealed a molecular profile particular to the genus *Brassica* [[8]](https://paperpile.com/c/bQ9QdB/aOe0b), where *n-*alkane C_29_, alcohol C_29_ nonacosane-15-ol and ketone C_29_ (nonacosane-15-one) were present. Leek yielded a profile containing ketone C_31_ (hentriacontane-16-one), alkane C_31_ and *n*-alcohols C_22_-C_26_ where C_28_ is dominant. This profile has already been suggested in several biomolecular archaeology papers [[9–12]](https://paperpile.com/c/bQ9QdB/mSYJy+JJ5IL+V5xQC+QNPKU). Of note, onion which is also of the genus *Allium* did not deliver a similar profile to leek.

# **Conclusion**

In conclusion, only plants of the genus *Brassica*, leeks and spinach have sufficiently specific profiles to be identified in the archaeological vessels. The lipid profiles specific to brassicae were identified in several archaeological samples in this study, alongside a lipid profile specific to leeks identified in one sample. However, no sample yielded a profile that could be attributed to the presence of spinach. However, it has been shown that sterols, the most specific compounds in spinach, are rapidly degraded, especially by cooking [[13]](https://paperpile.com/c/bQ9QdB/LVZT).

# **References**

1. [Craig OE, Saul H, Lucquin A, Nishida Y, Taché K, Clarke L, et al. Earliest evidence for the use of pottery. Nature. 2013;496: 351.](http://paperpile.com/b/bQ9QdB/YFwLM)

2. [Correa-Ascencio M, Evershed RP. High throughput screening of organic residues in archaeological potsherds using direct acidified methanol extraction. Analytical Methods. 2014;6: 1330–1340.](http://paperpile.com/b/bQ9QdB/v5ooG)

3. [Garnier N, Valamoti SM. Prehistoric wine-making at Dikili Tash (Northern Greece): Integrating residue analysis and archaeobotany. Journal of Archaeological Science. 2016;74: 195–206.](http://paperpile.com/b/bQ9QdB/IKjcW)

4. [Craig OE, Forster M, Andersen SH, Koch E, Crombé P, Milner NJ, et al. Molecular and isotopic demonstration of the processing of aquatic products in northern European prehistoric pottery . Archaeometry. 2007;49: 135–152.](http://paperpile.com/b/bQ9QdB/ShzKf)

5. [Dudd SN, Evershed RP. Direct demonstration of milk as an element of archaeological economies. Science. 1998;282: 1478–1481.](http://paperpile.com/b/bQ9QdB/kaI4Z)

6. [Watson AM. The Arab Agricultural Revolution and Its Diffusion, 700-1100. Journal of Economic History. 1974;34: 8–35.](http://paperpile.com/b/bQ9QdB/Bb7lu)

7. [Watson AM. Agricultural innovation in the early Islamic world: the diffusion of crops and farming techniques, 700-1100. University Press. 1983.](http://paperpile.com/b/bQ9QdB/Ajxax)

8. [Charters S, Evershed RP, Quye A, Blinkhorn PW, Reeves V. Simulation Experiments for Determining the Use of Ancient Pottery Vessels: the Behaviour of Epicuticular Leaf Wax During Boiling of a Leafy Vegetable. Journal of Archaeological Science. 1997;24: 1–7.](http://paperpile.com/b/bQ9QdB/aOe0b)

9. Evershed RP, Heron C, Charters S, Goad LJ. The survival of food residues: new methods of analysis, interpretation and application. Proceedings of the British Academy. 1992; 77(187): 2

10. [Evershed RP, Stott AW, Raven A, Dudd SN, Charters S, Leyden A. Formation of long-chain ketones in ancient pottery vessels by pyrolysis of acyl lipids. Tetrahedron Lett. 1995;36: 8875–8878.](http://paperpile.com/b/bQ9QdB/JJ5IL)

11. [Raven AM, van Bergen PF, Stott AW, Dudd SN, Evershed RP. Formation of long-chain ketones in archaeological pottery vessels by pyrolysis of acyl lipids. J Anal Appl Pyrolysis. 1997;40-41: 267–285.](http://paperpile.com/b/bQ9QdB/V5xQC)

12. [Rhee Y, Hlousek-Radojcic A, Ponsamuel J, Liu D, Post-Beittenmiller D. Epicuticular Wax Accumulation and Fatty Acid Elongation Activities Are Induced during Leaf Development of Leeks1. Plant Physiol. 1998;116: 901–911.](http://paperpile.com/b/bQ9QdB/QNPKU)

13. [Hammann S, Whittle M, Cramp LJE, Evershed RP. Cholesterol degradation in archaeological pottery mediated by fired clay and fatty acid pro-oxidants. Tetrahedron Letters. 2018. doi:](http://paperpile.com/b/bQ9QdB/LVZT)[10.1016/j.tetlet.2018.10.071](http://dx.doi.org/10.1016/j.tetlet.2018.10.071)
