# Supplementary material for: New insights into early medieval Islamic cuisine: Organic residue analysis of pottery from rural and urban Sicily
Source: PLoS One. 2021 Jun 9;16(6):e0252225. doi: 10.1371/journal.pone.0252225 (PMC8189454; doi:10.1371/journal.pone.0252225)
Supplement: S4 Text — (DOCX) [file pone.0252225.s004.docx]

**S4 Text. Summary of Faunal remains**

A summary of faunal remains identified at the sites studied in this research (Palazzo Bonagia [1,2]; the Gancia Church [1,2]; Castello San Pietro [3] and Casale San Pietro [3] . These are presented in terms of number of identified species (NISP) and percentage of number of identified species (%NISP) and reference to the original studies that analysed these samples is cited.

Table S3.1 Summary of faunal remains at Palazzo Bonagia (PB) reported as number of identified species (NISP) and % NISP

| **Species** | **NISP** | **%NISP** | **Reference to original study** |
| --- | --- | --- | --- |
| Caprines | 652 | 65.26 | [1,2] |
| Cattle | 207 | 20.74 |  |
| Pig | 8 | 0.8 |  |
| Chicken | 15 | 1.5 |  |
| Horse | 14 | 1.4 |  |
| Deer | 7 | 0.7 |  |
| Tuna | 16 | 1.6 |  |
| Dog | 21 | 2.1 |  |
| Cat | 3 | 0.3 |  |
| Rat | 2 | 0.2 |  |
| Total | 998 |  |  |

Table S3.2 Summary of faunal remains at the Gancia Church (GA) reported as number of identified species (NISP) and % NISP

| **Species** | **NISP** | **%NISP** | **Reference to original study** |
| --- | --- | --- | --- |
| Caprines | 2258 | 71.21 | [1,2] |
| Cattle | 404 | 12.74 |  |
| Pig | 51 | 1.61 |  |
| Chicken | 145 | 4.57 |  |
| Horse | 46 | 1.45 |  |
| Deer | 14 | 0.44 |  |
| Tuna | 88 | 2.77 |  |
| Other birds | 50 | 1.59 |  |
| Dog | 17 | 0.54 |  |
| Cat | 15 | 0.47 |  |
| Fox | 3 | 0.09 |  |
| Total | 3171 |  |  |

Table S3.3 Summary of faunal remains at Castello San Pietro (CSP) reported as number of identified species (NISP) and % NISP

| **Species** | **NISP** | **%NISP** | **Reference to original study** |
| --- | --- | --- | --- |
| Caprines | 231 | 60.4712 | [3] |
| Cattle | 47 | 12.30366 |  |
| Pig | 56 | 14.65969 |  |
| Chicken | 18 | 4.712042 |  |
| Horse | 1 | 0.26178 |  |
| Tuna | 10 | 2.617801 |  |
| Dog | 2 | 0.52356 |  |
| Cat | 3 | 0.78534 |  |
| Total | 382 |  |  |

Table S3.4 Summary of faunal remains at Casale San Pietro (CLESP) reported as number of identified species (NISP) and % NISP

| **Species** | **NISP** | **%NISP** | **Reference to original study** |
| --- | --- | --- | --- |
| Caprines | 69 | 35.9375 | [3] |
| Cattle | 23 | 11.97917 |  |
| Pig | 62 | 32.29167 |  |
| Chicken | 1 | 0.520833 |  |
| Horse | 14 | 7.291667 |  |
| Deer | 8 | 4.166667 |  |
| Tuna | 4 | 2.083333 |  |
| Dog | 21 | 10.9375 |  |
| Total | 192 |  |  |

1. Arcoleo L. Indagini archeozoologiche della Palermo antica: la Gancia, Palazzo Bonagia e Via Imera. Università degli Studi di Palermo. 2015. Available: https://iris.unipa.it/handle/10447/106536

2. Arcoleo L, Sineo L. Analisi archeozoologica di due contesti della città antica di Palermo: la gancia ei sili di via Imera (Palermo, IX-X secolo dC). Analisi archeozoologica di due contesti della città. 2014.

3. Aniceti V. Animals and their roles in the medieval society of Sicily: from Byzantine to Arabs and from Arabs to Norman/ Swabians. Doctor of Philosophy , The University of Sheffield . 2020.
