# Supplementary material for: New insights into early medieval Islamic cuisine: Organic residue analysis of pottery from rural and urban Sicily
Source: PLoS One. 2021 Jun 9;16(6):e0252225. doi: 10.1371/journal.pone.0252225 (PMC8189454; doi:10.1371/journal.pone.0252225)
Supplement: S1 Table — (DOCX) [file pone.0252225.s006.docx]

# S1 Table. Malic acid and tartaric acid quantities in vegetables.

Here the malic acid and tartaric acid quantities identified in a variety of vegetables from previously published studies are presented. These values, alongside values for different fruits published in [1], were used to produce Figure 7a in the main text.

**S1 Table. Malic acid and tartaric acid quantities in vegetables.** Values taken from previously published studies as shown in table. %TA = tartaric acid/ (tartaric + malic acid)

| **Category** | **Product** | **Reference** | **Yield tartaric acid (TA) (μg/g)** | **Yield malic acid (MA) (μg/g)** | **TA/MA** | **%TA** |
| --- | --- | --- | --- | --- | --- | --- |
| Vegetables | Spinach | [[2]](https://paperpile.com/c/gK3FZD/VqsV) | 0.00 | 13.00 | 0.00 | 0.00 |
| Vegetables | Eddoe | [[2]](https://paperpile.com/c/gK3FZD/VqsV) | 18.30 | 73.90 | 0.25 | 0.20 |
| Vegetables | Carrot | [[3]](https://paperpile.com/c/gK3FZD/jqFI) | 0.00 | 93.38 | 0.00 | 0.00 |
| Vegetables | Peas | [[3]](https://paperpile.com/c/gK3FZD/jqFI) | 0.00 | 15.56 | 0.00 | 0.00 |
| Vegetables | Carrot | [[4]](https://paperpile.com/c/gK3FZD/rqGb) | 1.80 | 236.00 | 0.01 | 0.01 |
| Vegetables | Carrot | [[4]](https://paperpile.com/c/gK3FZD/rqGb) | 2.00 | 524.00 | 0.00 | 0.00 |
| Vegetables | Carrot | [[4]](https://paperpile.com/c/gK3FZD/rqGb) | 2.70 | 502.00 | 0.01 | 0.01 |
| Vegetables | Carrot | [[4]](https://paperpile.com/c/gK3FZD/rqGb) | 5.50 | 327.00 | 0.02 | 0.02 |
| Vegetables | Carrot | [[4]](https://paperpile.com/c/gK3FZD/rqGb) | 1.90 | 305.00 | 0.01 | 0.01 |
| Vegetables | Carrot | [[4]](https://paperpile.com/c/gK3FZD/rqGb) | 1.80 | 275.00 | 0.01 | 0.01 |
| Vegetables | Carrot | [[5]](https://paperpile.com/c/gK3FZD/As42) | 0.01 | 0.59 | 0.01 | 0.01 |
| Vegetables | Carrot | [[5]](https://paperpile.com/c/gK3FZD/As42) | 0.01 | 0.59 | 0.01 | 0.01 |
| Vegetables | Carrot | [[5]](https://paperpile.com/c/gK3FZD/As42) | 0.01 | 0.58 | 0.01 | 0.01 |
| Vegetables | Carrot | [[5]](https://paperpile.com/c/gK3FZD/As42) | 0.01 | 0.58 | 0.01 | 0.01 |
| Vegetables | Cucumber | [[5]](https://paperpile.com/c/gK3FZD/As42) | 0.01 | 0.17 | 0.06 | 0.06 |
| Vegetables | Leek | [[4]](https://paperpile.com/c/gK3FZD/rqGb) | 0.00 | 192.00 | 0.00 | 0.00 |
| Vegetables | Leek | [[4]](https://paperpile.com/c/gK3FZD/rqGb) | 0.00 | 175.00 | 0.00 | 0.00 |
| Vegetables | Leek | [[4]](https://paperpile.com/c/gK3FZD/rqGb) | 0.00 | 155.00 | 0.00 | 0.00 |
| Vegetables | Chives | [[4]](https://paperpile.com/c/gK3FZD/rqGb) | 0.00 | 240.00 | 0.00 | 0.00 |
| Vegetables | Chinese cabbage | [[4]](https://paperpile.com/c/gK3FZD/rqGb) | 0.00 | 96.00 | 0.00 | 0.00 |
| Vegetables | Chinese cabbage | [[4]](https://paperpile.com/c/gK3FZD/rqGb) | 0.00 | 126.00 | 0.00 | 0.00 |
| Vegetables | Chinese cabbage | [[4]](https://paperpile.com/c/gK3FZD/rqGb) | 0.00 | 108.00 | 0.00 | 0.00 |
| Vegetables | Cauliflower | [[4]](https://paperpile.com/c/gK3FZD/rqGb) | 0.00 | 126.00 | 0.00 | 0.00 |
| Vegetables | Cauliflower | [[4]](https://paperpile.com/c/gK3FZD/rqGb) | 0.00 | 276.00 | 0.00 | 0.00 |
| Vegetables | Cauliflower | [[4]](https://paperpile.com/c/gK3FZD/rqGb) | 0.00 | 239.00 | 0.00 | 0.00 |
| Vegetables | Kale | [[4]](https://paperpile.com/c/gK3FZD/rqGb) | 0.00 | 174.00 | 0.00 | 0.00 |
| Vegetables | Kale | [[4]](https://paperpile.com/c/gK3FZD/rqGb) | 0.00 | 158.00 | 0.00 | 0.00 |
| Vegetables | Brussels sprouts | [[4]](https://paperpile.com/c/gK3FZD/rqGb) | 0.00 | 189.00 | 0.00 | 0.00 |
| Vegetables | Brussels sprouts | [[4]](https://paperpile.com/c/gK3FZD/rqGb) | 0.00 | 262.00 | 0.00 | 0.00 |
| Vegetables | Brussels sprouts | [[4]](https://paperpile.com/c/gK3FZD/rqGb) | 0.00 | 410.00 | 0.00 | 0.00 |
| Vegetables | Red cabbage | [[4]](https://paperpile.com/c/gK3FZD/rqGb) | 0.00 | 75.00 | 0.00 | 0.00 |
| Vegetables | Red cabbage | [[4]](https://paperpile.com/c/gK3FZD/rqGb) | 0.00 | 63.00 | 0.00 | 0.00 |
| Vegetables | Red cabbage | [[4]](https://paperpile.com/c/gK3FZD/rqGb) | 0.00 | 71.00 | 0.00 | 0.00 |
| Vegetables | White cabbage | [[4]](https://paperpile.com/c/gK3FZD/rqGb) | 0.00 | 107.00 | 0.00 | 0.00 |
| Vegetables | White cabbage | [[4]](https://paperpile.com/c/gK3FZD/rqGb) | 0.00 | 96.00 | 0.00 | 0.00 |
| Vegetables | White cabbage | [[4]](https://paperpile.com/c/gK3FZD/rqGb) | 0.00 | 84.00 | 0.00 | 0.00 |
| Vegetables | Savoy cabbage | [[4]](https://paperpile.com/c/gK3FZD/rqGb) | 0.00 | 93.00 | 0.00 | 0.00 |
| Vegetables | Savoy cabbage | [[4]](https://paperpile.com/c/gK3FZD/rqGb) | 0.00 | 105.00 | 0.00 | 0.00 |
| Vegetables | Lettuce | [[4]](https://paperpile.com/c/gK3FZD/rqGb) | 5.60 | 168.00 | 0.03 | 0.03 |
| Vegetables | Lettuce | [[4]](https://paperpile.com/c/gK3FZD/rqGb) | 8.10 | 243.00 | 0.03 | 0.03 |
| Vegetables | Lettuce | [[4]](https://paperpile.com/c/gK3FZD/rqGb) | 10.70 | 92.00 | 0.12 | 0.10 |
| Vegetables | Endive | [[4]](https://paperpile.com/c/gK3FZD/rqGb) | 7.00 | 179.00 | 0.04 | 0.04 |
| Vegetables | Endive | [[4]](https://paperpile.com/c/gK3FZD/rqGb) | 6.00 | 155.00 | 0.04 | 0.04 |
| Vegetables | Chicory | [[4]](https://paperpile.com/c/gK3FZD/rqGb) | 6.10 | 258.00 | 0.02 | 0.02 |
| Vegetables | Chicory | [[4]](https://paperpile.com/c/gK3FZD/rqGb) | 12.50 | 185.00 | 0.07 | 0.06 |
| Vegetables | Celery | [[4]](https://paperpile.com/c/gK3FZD/rqGb) | 0.00 | 495.00 | 0.00 | 0.00 |
| Vegetables | Celery | [[4]](https://paperpile.com/c/gK3FZD/rqGb) | 0.00 | 474.00 | 0.00 | 0.00 |
| Vegetables | Celery | [[4]](https://paperpile.com/c/gK3FZD/rqGb) | 0.60 | 447.00 | 0.00 | 0.00 |
| Vegetables | Celery | [[4]](https://paperpile.com/c/gK3FZD/rqGb) | 2.30 | 226.00 | 0.01 | 0.01 |
| Vegetables | Celery | [[4]](https://paperpile.com/c/gK3FZD/rqGb) | 0.00 | 397.00 | 0.00 | 0.00 |
| Vegetables | Beets | [[4]](https://paperpile.com/c/gK3FZD/rqGb) | 0.00 | 22.00 | 0.00 | 0.00 |
| Vegetables | Beets | [[4]](https://paperpile.com/c/gK3FZD/rqGb) | 0.00 | 18.00 | 0.00 | 0.00 |
| Vegetables | Beets | [[4]](https://paperpile.com/c/gK3FZD/rqGb) | 0.00 | 17.00 | 0.00 | 0.00 |
| Vegetables | Spinach | [[4]](https://paperpile.com/c/gK3FZD/rqGb) | 0.00 | 37.00 | 0.00 | 0.00 |
| Vegetables | Spinach | [[4]](https://paperpile.com/c/gK3FZD/rqGb) | 0.00 | 47.00 | 0.00 | 0.00 |
| Vegetables | Spinach | [[4]](https://paperpile.com/c/gK3FZD/rqGb) | 0.00 | 36.00 | 0.00 | 0.00 |
| Vegetables | Spinach | [[4]](https://paperpile.com/c/gK3FZD/rqGb) | 0.00 | 64.00 | 0.00 | 0.00 |
| Onion | Onion | [[6]](https://paperpile.com/c/gK3FZD/1qoq) | 13.93 | 60.43 | 0.23 | 0.19 |
| Onion | Onion | [[6]](https://paperpile.com/c/gK3FZD/1qoq) | 6.10 | 66.33 | 0.09 | 0.08 |
| Onion | Onion | [[6]](https://paperpile.com/c/gK3FZD/1qoq) | 16.15 | 61.85 | 0.26 | 0.21 |
| Onion | Onion | [[6]](https://paperpile.com/c/gK3FZD/1qoq) | 25.88 | 57.61 | 0.45 | 0.31 |
| Onion | Onion | [[6]](https://paperpile.com/c/gK3FZD/1qoq) | 12.02 | 78.94 | 0.15 | 0.13 |
| Onion | Onion | [[4]](https://paperpile.com/c/gK3FZD/rqGb) | 0.00 | 194.00 | 0.00 | 0.00 |
| Onion | Onion | [[4]](https://paperpile.com/c/gK3FZD/rqGb) | 0.00 | 196.00 | 0.00 | 0.00 |
| Onion | Onion | [[4]](https://paperpile.com/c/gK3FZD/rqGb) | 0.00 | 155.00 | 0.00 | 0.00 |
| Onion | Onion | [[4]](https://paperpile.com/c/gK3FZD/rqGb) | 0.00 | 184.00 | 0.00 | 0.00 |
| Onion | Onion | [[7]](https://paperpile.com/c/gK3FZD/Qsuu) | 8.90 | 47.80 | 0.19 | 0.16 |
| Onion | Onion | [[7]](https://paperpile.com/c/gK3FZD/Qsuu) | 23.30 | 49.30 | 0.47 | 0.32 |
| Onion | Onion | [[7]](https://paperpile.com/c/gK3FZD/Qsuu) | 19.20 | 31.10 | 0.62 | 0.38 |
| Onion | Onion | [[7]](https://paperpile.com/c/gK3FZD/Qsuu) | 15.50 | 50.30 | 0.31 | 0.24 |
| Onion | Onion | [[7]](https://paperpile.com/c/gK3FZD/Qsuu) | 14.00 | 34.30 | 0.41 | 0.29 |
| Onion | Onion | [[7]](https://paperpile.com/c/gK3FZD/Qsuu) | 25.20 | 44.70 | 0.56 | 0.36 |

1. Drieu L, Orecchioni P, Capelli C, Meo A, Lundy J, Sacco V, et al. Chemical evidence for the persistence of wine production and trade in Early Medieval Islamic Sicily. Proc Natl Acad Sci U S A. 2021;118. doi:10.1073/pnas.2017983118

2. [Askar A, El-Samahy SK, Abd El-Fadeel MG. Organic acids and free amino acids in some Egyptian fruits and vegetables. Nahrung. 1982;26: K7–K10. doi:](http://paperpile.com/b/gK3FZD/VqsV)[10.1002/food.19820260140](http://dx.doi.org/10.1002/food.19820260140)

3. [Mabesa LD, Baldwin RE, Garner GB. Non-volatile Organic Acid Profiles of Peas and Carrots Cooked by Microwaves. J Food Prot. 1979;42: 385–388. doi:](http://paperpile.com/b/gK3FZD/jqFI)[10.4315/0362-028X-42.5.385](http://dx.doi.org/10.4315/0362-028X-42.5.385)

4. [Ruhl I, Herrmann K. Organische Säuren der Gemüsearten. I. Kohlarten, Blatt- und Zwiebelgemüse sowie Möhren und Sellerie. Zeitschrift für Lebensmittel-Untersuchung und Forschung. 1985;180: 215–220. doi:](http://paperpile.com/b/gK3FZD/rqGb)[10.1007/BF01027268](http://dx.doi.org/10.1007/BF01027268)

5. [Morvai M, Molnár-Perl I, Knausz D. Simultaneous gas—liquid chromatographic determination of sugars and organic acids as trimethylsilyl derivatives in vegetables and strawberries. J Chromatogr A. 1991;552: 337–344. doi:](http://paperpile.com/b/gK3FZD/As42)[10.1016/S0021-9673(01)95950-3](http://dx.doi.org/10.1016/S0021-9673(01)95950-3)

6. [Liguori L, Califano R, Albanese D, Raimo F, Crescitelli A, Di Matteo M. Chemical Composition and Antioxidant Properties of Five White Onion (Allium cepa L.) Landraces. J Food Qual. 2017. doi:](http://paperpile.com/b/gK3FZD/1qoq)[10.1155/2017/6873651](http://dx.doi.org/10.1155/2017/6873651)

7. [Rodriguez Galdon B, Tascon Rodriguez C, Rodríguez Rodríguez E, Diaz Romero C. Organic acid contents in onion cultivars (Allium cepa L.). J Agric Food Chem. 2008;56: 6512–6519.](http://paperpile.com/b/gK3FZD/Qsuu)
